# Adaptive reduction of male gamete number in the selfing plant *Arabidopsis thaliana*

**DOI:** 10.1038/s41467-020-16679-7

**Published:** 2020-06-08

**Authors:** Takashi Tsuchimatsu, Hiroyuki Kakui, Misako Yamazaki, Cindy Marona, Hiroki Tsutsui, Afif Hedhly, Dazhe Meng, Yutaka Sato, Thomas Städler, Ueli Grossniklaus, Masahiro M. Kanaoka, Michael Lenhard, Magnus Nordborg, Kentaro K. Shimizu

**Affiliations:** 1 Department of Evolutionary Biology and Environmental Studies & Zurich-Basel Plant Science Center, University of Zurich, 8057 Zurich, Switzerland; 2 Department of Plant and Microbial Biology & Zurich-Basel Plant Science Center, University of Zurich, 8008 Zurich, Switzerland; 3 Gregor Mendel Institute, Austrian Academy of Sciences, Vienna BioCenter, A-1030 Vienna, Austria; 4 Department of Biology, Chiba University, Chiba, 263-8522 Japan; 5 Department of Biological Sciences, University of Tokyo, Tokyo, 113-0033 Japan; 6 Kihara Institute for Biological Research, Yokohama City University, Yokohama, 244-0813 Japan; 7 Graduate School of Science and Technology, Niigata University, Niigata, 950-2181 Japan; 8 Institute for Biochemistry and Biology, University of Potsdam, 14476 Potsdam, Germany; 9 Graduate School of Science, Nagoya University, Nagoya, 464-8602 Japan; 10 JST ERATO Higashiyama Live-Holonics Project, Nagoya University, Nagoya, 464-8602 Japan; 11 Molecular and Computational Biology, University of Southern California, Los Angeles, CA 90089-0371 USA; 12 Department of Genomics and Evolutionary Biology, National Institute of Genetics, Mishima, Shizuoka 411-8540 Japan; 13 Institute of Integrative Biology, ETH Zurich, 8092 Zurich, Switzerland

**Keywords:** Genetic variation, Genome-wide association studies, Pollen

## Abstract

The number of male gametes is critical for reproductive success and varies between and within species. The evolutionary reduction of the number of pollen grains encompassing the male gametes is widespread in selfing plants. Here, we employ genome-wide association study (GWAS) to identify underlying loci and to assess the molecular signatures of selection on pollen number-associated loci in the predominantly selfing plant *Arabidopsis thaliana*. Regions of strong association with pollen number are enriched for signatures of selection, indicating polygenic selection. We isolate the gene *REDUCED POLLEN NUMBER1* (*RDP1*) at the locus with the strongest association. We validate its effect using a quantitative complementation test with CRISPR/Cas9-generated null mutants in nonstandard wild accessions. In contrast to pleiotropic null mutants, only pollen numbers are significantly affected by natural allelic variants. These data support theoretical predictions that reduced investment in male gametes is advantageous in predominantly selfing species.

## Abstract (web summary)

Reduction of pollen grain number is widespread in selfing plants, but the determining gene is unknown. Here, the authors show that a ribosome-biogenesis factor encoding gene *RDP1* is responsible for adaptive reduction of male gamete number in *Arabidopsis thaliana*.

## Introduction

Male gamete numbers, reflected by pollen grain (each containing two sperm cells) numbers in seed plants and sperm numbers in animals, have been studied extensively from agricultural, medical, and evolutionary viewpoints^1–9^. Evolutionary theory predicts that the breeding system could act as a major selective force on male gamete numbers. In highly promiscuous outcrossing species, a large number of male gametes should be produced because of male–male gamete competition, so reduced male gamete numbers are considered to be deleterious^1^. In contrast, they may be advantageous at lower outcrossing rates because of the high cost of their production, decreasing fitness. In an agricultural context, low pollen number may have been selected during domestication^10^, but may serve as a barrier for hybrid breeding of wheat and other species^11,12^. In flowering plants, the transition from an outcrossing to a selfing breeding system through loss of self-incompatibility is one of the most prevalent evolutionary trends^6,13^. Selfing populations or species generally show lower pollen grain numbers per flower (hereafter, pollen number) as well as reduced flower size. There has been a sustained debate on whether the reduced pollen number is a result of adaptive evolution or the accumulation of deleterious mutations owing to reduced selection^6,14,15^, but little was known about the genetic basis of pollen number variation to assess molecular signatures of selection on it.

To unravel the genetic basis of quantitative natural variation in pollen number, we here focus on the predominantly selfing plant *Arabidopsis thaliana*^6,16^. Studies have shown that the evolution of predominant selfing in *A. thaliana* occurred much more recently than its evolutionary divergence from outcrossing relatives^6,16,17^. Thus, in addition to fixed, genetically based differences from these outcrossing relatives, we expect that variation with regard to pollen number may still be segregating among current accessions. By harnessing the genetic and genomic resources available in *A. thaliana*, we conduct a genome-wide association study on pollen number variation. We find that natural variants of the *RDP1* gene confer variation in pollen number without detectable pleiotropy. Signatures of selection at the top genome-wide association study (GWAS) peaks, including the *RDP1* locus, support the theoretical prediction that reduced investment in male gametes should provide an advantage in selfing species.

## Results

### Genome-wide association study and signatures of selection

**Fig. 1:**
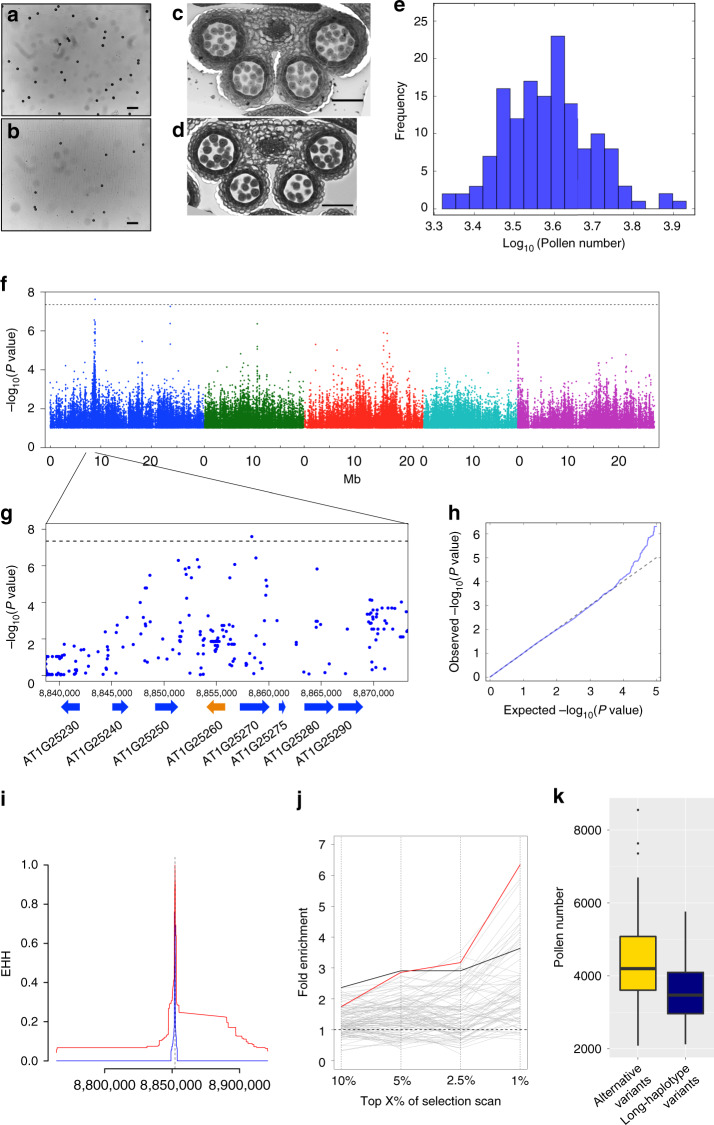
Genome-wide association study of pollen number variation in *Arabidopsis thaliana*. **a**, **b** Pollen grains of higher pollen number accession Bor-4 (**a**) and lower pollen number accession Mz-0 (**b**) mounted on glass slides for counting^52^. Scale bars = 100 μm. **c**, **d** Histological sections of Bor-4 (**c**) and Mz-0 (**d**) stamens. At least three independent observations showed similar results (**a**–**d**). Scale bars = 50 μm. **e** Distribution of pollen number variation across 144 natural accessions. **f** Manhattan plot of the genome-wide association study (GWAS). **g** Closer view of the region around the significant GWAS peak on chromosome 1 with gene models and coordinates. **f**, **g** SNPs with minor allele frequency > 0.15 are shown; horizontal dashed lines indicate the nominal *P* < 0.05 threshold after Bonferroni correction. **h** Quantile–quantile plot of the GWAS. **i** Extended haplotype homozygosity (EHH) detected in the *RDP1* genomic region. Red and blue lines correspond to the long haplotype and alternative variants, respectively. **j** Signatures of selection at pollen number-associated loci. Each line indicates a phenotype (red: pollen number, black: ovule number, gray: 107 phenotypes^19^). The *x* axis quantifies the extreme tails of the integrated haplotype score (iHS) statistic. The pollen and ovule GWAS show significant enrichment (permutation test, *P* < 0.05 cutoff for all iHS statistical tails; Supplementary Fig. 3). **k** Accessions with the long-haplotype variants (defined by SNP 1-8852112) generally showed lower pollen number (*P* = 2.152 × 10^–6^, two-sided *t* test; population structure-corrected GWAS *P* = 2.95 × 10^–6^). Boxplots show center line: median; box limits: upper and lower quartiles; whiskers: not >1.5 times the interquartile range; dots: outliers. Source data underlying **e**, **j** and **k** are provided as a Source Data file.

To examine variability in pollen number on a species-wide scale, we determined pollen number per flower in 144 natural *A. thaliana* accessions (Fig. 1a–d; Supplementary Table 1 and Supplementary Data 1) and found approximately fourfold variation (average ~4000) (Fig. 1e). Histological sections of stamens from representative accessions confirmed pollen number variation among accessions (Fig. 1c, d). We also measured the number of ovules per flower (Supplementary Table 2). We did not find significant correlations between numbers of pollen grains and ovules (*P* = 0.5164), although negative correlations have often been reported in between-species comparisons, as expected on theoretical grounds owing to trade-offs in resource allocation to male versus female function^14,18^. Furthermore, we found that pollen number per flower was not significantly correlated with any of the 107 published phenotypes of flowering, defense-related, ionomic, and developmental traits (Supplementary Table 3; Supplementary Note 1)^19^, nor with climate variables, geographic location, or *S*-haplogroups across the 144 accessions (Supplementary Tables 4 and 5, Supplementary Fig. 1)^20,21^. These data suggest that variation in pollen number is largely independent of other traits.

To evaluate genome-wide signatures of natural selection on loci associated with gamete numbers, we first performed GWAS for pollen and ovule numbers using a genome-wide single-nucleotide polymorphism (SNP) data set for these lines, which was obtained by imputation based on genome-wide resequencing data and 250 k SNP data (Fig. 1f, h; Supplementary Fig. 2)^22^. In total, 68 peaks of association were identified (10-kb windows having SNPs with *P* < 10^–4^), although only one pollen number-associated peak remained significant after Bonferroni correction. Focusing on the identified GWAS peaks, we performed an enrichment analysis to ask whether pollen and ovule number-associated peaks are enriched in long-haplotype regions, which could be owing to partial or ongoing sweeps of segregating polymorphisms^23–25^. To identify long-haplotype regions, we first calculated the extended haplotype homozygosity (EHH), which measures decay of haplotypes that carry a specified core allele as a function of distance^23^. We then obtained the integrated haplotype score (iHS) statistic for each SNP, which compares EHH between two alleles of the SNP by controlling for the allele frequency of each SNP^23^. We found that 10-kb windows including pollen number-associated loci were significantly enriched in extreme iHS tails (*P* < 0.05, permutation test; Fig. 1i, j; Supplementary Fig. 3). These loci showed generally high iHS scores, and two of the top five GWAS peaks were outliers of the genome-wide iHS distribution (Supplementary Table 6). The enrichment was robust to changes in sample composition, allele frequency cutoffs, and the use of windows (Supplementary Figs. 4–6; see Supplementary Note 2 for details). Ovule number also showed enrichment, albeit less than pollen number (Fig. 1j). In principle, the iHS enrichment could be confounded by recombination rate and the accuracy of imputation. To deal with such potential confounding factors, we compared these results with the results of an iHS enrichment analysis for GWAS peaks (*P* < 0.0001) for 107 other phenotypes, as these confounding factors should also influence the enrichment for other traits. We found that the iHS enrichment for pollen number GWAS peaks (*P* = 0.002 for the top 1% iHS tail; Supplementary Fig. 3) was among the highest, compared with that for many known adaptive traits included in the 107 phenotypes, such as leaf number at flowering time and resistance to *Pseudomonas* pathogens^19,25^ (Supplementary Table 7). In addition, iHS enrichment of the ovule number GWAS peaks was also significant (*P* = 0.030 for the top 1% iHS tail; Supplementary Fig. 3). These enrichments support polygenic selection on a considerable number of loci associated with male and female gamete numbers throughout the genome.

### Isolation of the *REDUCED POLLEN NUMBER1* gene

To further understand the molecular basis of pollen number variation and to examine the nature of the putative targets of selection, we tried to identify the genes underlying pollen number variation; however, the top five peaks of association did not contain any genes with known functions in early stamen or pollen development. To obtain experimental evidence concerning genes underlying pollen number variation, we conducted functional analyses of the genes under the highest pollen number GWAS peak, which explains ~20% of the total phenotypic variance between accessions and satisfies the criterion for genome-wide significance (–log_10_ *P* = 7.60). This region is of particular interest because it also satisfies the criteria for genome-wide significance of the iHS statistic (*P* = 0.0149; Fig. 1i, Supplementary Fig. 7), suggesting a selective sweep. To test whether the signature of selection in this region might be owing to traits other than pollen number, we examined whether there is an association signal for any of the 107 published phenotypes, ovule number, or variants showing climatic correlations^19,20^. In the 10-kb window including the SNP of the highest GWAS score for pollen number, we found no genotype–phenotype associations below *P* < 10^–5^ or climate–SNP correlations below an empirical *P* < 0.01, i.e., there is no significant evidence for selection on traits other than pollen number in this region. We also found that accessions with the long-haplotype variants produced lower pollen numbers than those with alternative haplotype variants (*P* = 2.152 × 10^–6^, *t* test; population structure-corrected GWAS *P* = 2.95 × 10^–6^; Fig. 1k), as expected if this haplotype was under selection for reduced pollen number.

**Fig. 2:**
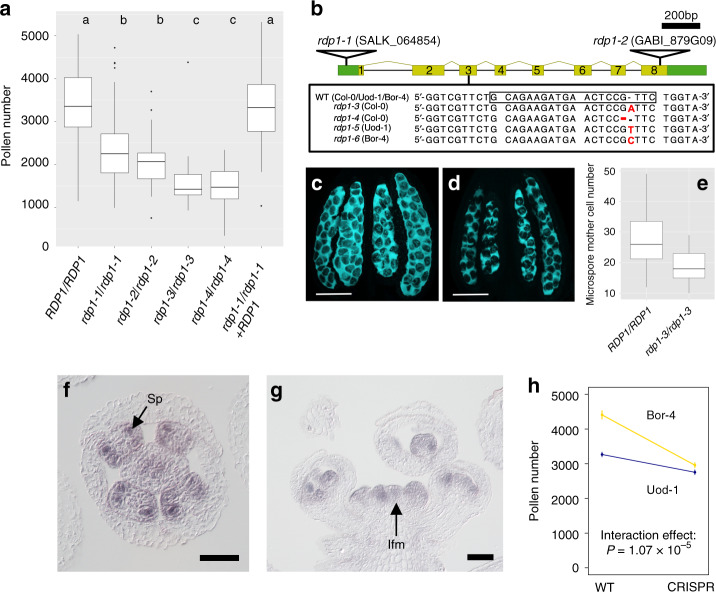
Functional characterization of the *RDP1* gene. **a** Pollen number differences between four homozygous mutants (*rdp1-1/rdp1-1*, *rdp1-2/rdp1-2*, *rdp1-3/rdp1-3*, and *rdp1-4/rdp1-4*), wild-type (*RDP1/RDP1*), and the complemented lines (*rdp1-1*/*rdp1-1* + *RDP1*). Letters a, b, c indicate significant differences, determined by nested analysis of variance (ANOVA) and post hoc Tukey test; *P* < 0.05 (See Supplementary Table 8 for *P* values). Numbers of flowers pollen-counted (left to right, as plotted): *N* = 132, 45, 40, 60, 135, 411. **b** Schematic structure of the *RDP1* gene. Untranslated regions (green boxes), coding regions (yellow boxes), introns (bars), two T-DNA insertion mutants (*rdp1-1*, *rdp1-2*; open triangles), and four CRISPR/Cas9-based frameshift mutants (*rdp1-3* and *rdp1-4* in Col-0, *rdp1-5* in Uod-1, and *rdp1-6* in Bor-4 backgrounds, respectively). The box in the wild-type sequences indicates the target site for CRISPR/Cas9, and red letters in mutants show the indel mutation. **c**, **d** Representative microsporocyte images of *RDP1/RDP1* (**c**) and *rdp1-3/rdp1-3* (**d**) stained with aniline blue. Dark spots indicate microsporocytes. Summary of observation is depicted in **e**. **e** The number of microsporocytes was significantly lower in *rdp1-3/rdp1-3* than in the wild type (Wilcoxon rank sum test, *P* = 2.82e–06). Numbers of anthers counted (left to right, as plotted): *N* = 40 and 50. **f**, **g** In situ hybridization for *RDP1*. Similar expression patterns were observed in four (**f**) and two (**g**) individuals. **f** Stage 8 floral cross section; expression is detected in sporogenous cells (Sp). **g** Inflorescence longitudinal section; expression is observed in the inflorescence meristem (Ifm) and young flowers. **h** Pollen number of wild type and null mutants in the Uod-1 (blue) and Bor-4 (yellow) accessions. Dots and error bars indicate means and standard errors of means, respectively. The mutation in Bor-4 caused a stronger reduction in pollen number than that in Uod-1 (ANOVA interaction effect *P* = 1.07 × 10^–5^, one-sided test). Boxplots **a**, **e** show center line: median; box limits: upper and lower quartiles; whiskers: not >1.5 times the interquartile range; dots: outliers. Scale bars: 50 μm. Source data underlying **a**, **e** and **h** are provided as a Source Data file.

Of the three genes in this chromosomal region with the highest GWAS scores (AT1G25250, AT1G25260, and AT1G25270; Fig. 1g), the expression level of AT1G25260, a gene of unknown function, was much higher in flower buds than that of the other two genes (Supplementary Fig. 8). Therefore, we obtained two T-DNA insertion mutants of AT1G25260 in the standard Col-0 accession from the Nottingham *Arabidopsis* Stock Centre^26^. These mutants showed a 32% reduction in pollen number (Fig. 2a; Supplementary Table 8). We hereafter refer to AT1G25260 as *REDUCED POLLEN NUMBER1* (*RDP1*). Because both *rdp1-1* (insertion in the 5′ UTR) and *rdp1-2* (insertion at the end of the coding sequence) (Fig. 2b) homozygotes showed low levels of expression, they are likely to be hypomorphic mutants (Supplementary Fig. 8). We generated two amorphic (null) frameshift mutants of *RDP1* (*rdp1-3* and *rdp1-4*) using the CRISPR/Cas9 system^27,28^ in the Col-0 background. These mutants indeed showed an even greater reduction in pollen number, but still produced about half the number of pollen grains of the corresponding wild type (53% for *rdp1-3*; Fig. 2a), suggesting a quantitative nature of the effect of *RDP1*. Pollen size was slightly increased, in agreement with the well-known negative relationship between pollen number and size, even within the same genotype (Supplementary Fig. 9; Supplementary Table 9)^14^. The mutant phenotype was complemented by transforming a 4.3-kb genomic fragment of the Col-0 accession encompassing *RDP1* (Fig. 2a, Supplementary Fig. 9). In contrast to *RDP1* mutants, CRISPR/Cas9 induced null mutants in AT1G25250 or AT1G25270 did not result in any significant change in pollen number (Supplementary Fig. 10). The phenotype of four independent mutants of *RDP1* together with successful complementation using the wild-type allele thus demonstrated that *RDP1* is involved in the control of pollen number.

Based on phylogenetic analysis, RDP1 is a putative homolog of the yeast mRNA turnover 4 protein (Mrt4p) (Supplementary Figs. 11 and 12). The *MRT4* gene is nonessential in yeast but null mutants show a phenotype of slightly slower growth. The Mrt4p shares similarity with the ribosome P0 protein and is necessary for the assembly of the P0 protein into the ribosome. The human ribosome P0 gene is reported to have an extra-ribosomal function in cancer by modulating cell proliferation^29,30^. During anther development, sporogenous cells first divide and differentiate into microsporocytes^31^. Following meiosis of the microsporocyte, four microspores are formed, each of which undergoes two mitotic divisions to form a mature pollen grain containing the male gametes. The null *rdp1*-*3* mutant produced fewer microsporocytes than the wild type (Fig. 2c–e), indicating a reduction in cell numbers before meiosis. Consistent with this, in situ mRNA hybridization experiments detected strong expression of *RDP1* in sporogenous cells and the microsporocytes derived from them, but not in microspores (Fig. 2f, g; Supplementary Fig. 13). *RDP1* was also expressed in other proliferating cells, including those in inflorescences, floral meristematic regions, and ovules (Supplementary Figs. 8 and 13), supporting a more widespread role in proliferating cell types with putatively high demands for ribosome biogenesis^32^. Furthermore, fusing the *RDP1* promoter to the *uidA* reporter gene encoding β-glucuronidase (GUS) to assess its activity confirmed the *RDP1* expression pattern in stamens (Supplementary Fig. 14) and demonstrated marked expression in root tips and young leaf primordia during the vegetative phase; these data are supported by quantitative reverse transcription PCR experiments (Supplementary Fig. 8b). Consistent with *RDP1* expression in proliferating tissues, the *rdp1-3* null mutant showed pleiotropic phenotypes, including slower vegetative growth and reduced ovule numbers per flower (Supplementary Fig. 15). Because these pleiotropic phenotypes would be deleterious in natural environments, these data indicate that natural alleles of *RDP1* are not null variants (see below). In summary, these data suggest that *RDP1* is required in proliferating *A. thaliana* cells, yet the natural variants we have identified predominantly affect the proliferation of sporogenous cells in the anthers, consistent with the function of its yeast homolog in cell proliferation.

### Natural variants of *RDP1* confer pollen number variation

It has been difficult to experimentally determine whether a particular gene has natural alleles with subtle phenotypic effects on quantitative traits^33^. When allelic effects are subtle, transgenic analysis of natural alleles is not sufficiently powerful because the phenotypes of transgenic *A. thaliana* plants tend to be highly variable as a result of the variation between lines, e.g., owing to different transgene insertion sites. In contrast, a quantitative complementation test can identify responsible genes by testing the effect of natural alleles in a heterozygous state with a null allele if the effects of other loci are small, although this may be confounded by polygenic effects in the genetic background^33,34^. To conduct such quantitative complementation, we took advantage of the CRISPR/Cas9 technique to generate frameshift null alleles in nonstandard natural accessions, in which no prior mutant was available.

**Fig. 3:**
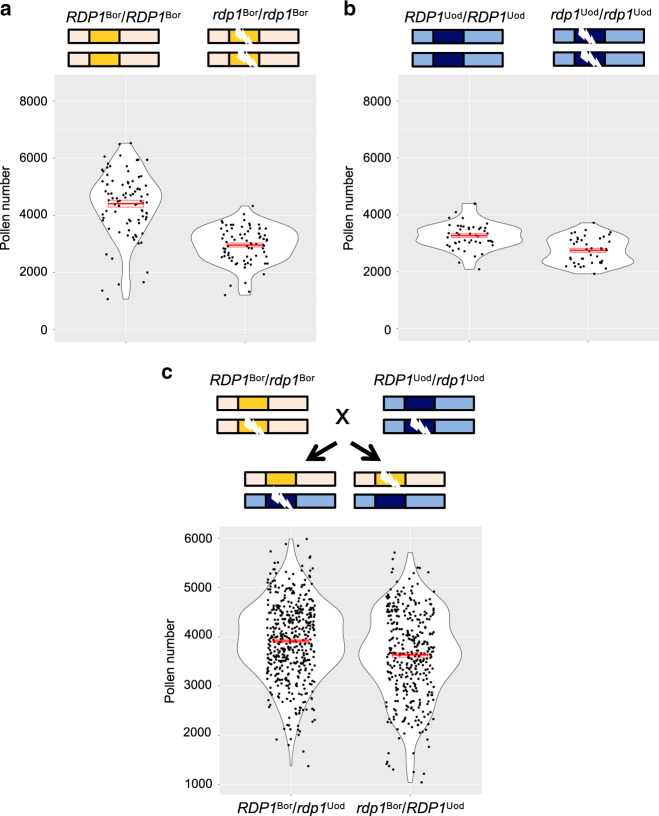
Quantitative complementation test of the *RDP1* gene. Violin plots with means and standard errors of means indicated by red bold bars and boxes, respectively. **a**, **b** Pollen number differences between wild-type and homozygous plants of a frameshift allele generated by the CRISPR/Cas9 technique in the Bor-4 background (**a** Numbers of flowers pollen-counted: *RDP1*^Bor^*/RDP1*^Bor^, *N* = 89; *rdp1*^Bor^*/rdp1*^Bor^, *N* = 77) and in the Uod-1 background (**b** Numbers of flowers pollen-counted: *RDP1*^Uod^*/RDP1*^Uod^, *N* = 47; *rdp1*^Uod^*/rdp1*^Uod^, *N* = 43) (same data sets with Fig. 2h). **c** The difference in the effect on pollen number by two natural alleles, *RDP1*^Bor^ and *RDP1*^Uod^. Pollen number of plants with *RDP1*^Uod^ was significantly lower than that of plants with *RDP1*^Bor^ (nested analysis of variance; *P* = 4.85 × 10^–8^; Numbers of flowers pollen-counted: *RDP1*^Bor^*/rdp1*^Uod^, *N* = 468 from 26 individuals; *rdp1*^Bor^*/RDP1*^Uod^, *N* = 368 from 20 individuals). The two alleles were compared in the heterozygous state with a frameshift CRISPR/Cas9 allele, with otherwise identical genetic backgrounds. F_1_ plants were obtained from the cross of two heterozygotes, *RDP1*^Bor^*/rdp1*^Bor^ and *RDP1*^Uod^*/rdp1*^Uod^. Source data are provided as a Source Data file.

We used Bor-4 and Uod-1, which have high and low pollen number phenotypes, respectively (Fig. 2b; Fig. 3). There were a number of sequence differences between the two accessions in the region encompassing the *RDP1* gene from 777 bp upstream of the start codon to 643 bp downstream of the stop codon. We found one non-synonymous and six synonymous substitutions in the coding region, and 62 substitutions and six indel mutations in the non-coding region (Supplementary Fig. 16). Yet, both accessions did not reveal obvious loss-of-function mutations (Supplementary Fig. 16), and *rdp1* CRISPR null mutants of each accession showed reduced pollen number compared with the corresponding wild type (*P* < 2.2 × 10^–16^ for Bor-4, *P* = 9.84 × 10^–7^ for Uod-1; Fig. 3a, b; Fig. 2h). These results show that both of the naturally occurring variants of the *RDP1* gene are not null mutants but rather encode a functional protein. Disruption of *RDP1* had a stronger effect on pollen number in Bor-4 than in Uod-1 (analysis of variance (ANOVA) interaction effect *P* = 1.07 × 10^–5^; Fig. 2h). This finding supports the notion that the Bor-4 allele has a stronger promotive effect on pollen number than the Uod-1 allele, although other loci in the genetic backgrounds of Bor-4 and Uod-1 may contribute to this difference through epistasis.

To test the allelic effect of *RDP1*, we utilized a quantitative complementation test that controls for genetic background (Fig. 3). Among F_1_ plants obtained by crossing heterozygotes for the frameshift mutation in each genetic background, we compared two genotypes: *RDP1*^Bor^*/rdp1*^Uod^ vs. *rdp1*^Bor^*/RDP1*^Uod^. These F_1_ genotypes are identical except for the differences at *RDP1*, where they both carry a frameshift allele but differ with respect to the functional allele; because of the crossing design, any independently segregating off-target effects, resulting from CRISPR/Cas9 mutagenesis would be equally distributed between the two genotype cohorts of interest. We found that pollen number in plants with *RDP1*^Uod^ was significantly lower than in plants with *RDP1*^Bor^ (nested ANOVA, 468 flowers from 26 individuals of *RDP1*^Bor^/*rdp1*^Uod^ and 368 flowers from 20 individuals of *rdp1*^Bor^*/RDP1*^Uod^, *P* = 4.85 × 10^–8^; Fig. 3c). The significant difference cannot be attributed to stochastic individual differences, because the significant difference between plants bearing functional *RDP1*^Bor^ and *RDP1*^Uod^ alleles was also observed in an individual-based test using averaged data of each individual separately (*P* = 0.0331). Thus, in an otherwise identical genetic background, the respective functional *RDP1* haplotypes cause differences in pollen number. We also measured rosette leaf size, flowering date, ovule number, dry weight, and seed weight of plants in the two cohorts, but none of these traits showed significant differences between *RDP1*^Bor^/*rdp1*^Uod^ and *rdp1*^Bor^/*RDP1*^Uod^ (Supplementary Fig. 17). Thus, in contrast to the experimentally generated null mutants that showed pleiotropic growth defects, these results indicate that natural allelic differences at *RDP1* affect pollen number in the absence of any detectable deleterious pleiotropy. This finding is also supported by no genotype–phenotype associations for other traits, as described above.

## Discussion

We here isolated the *RDP1* gene underlying natural variation in male gamete numbers. Our study provides evidence for polygenic selection on pollen number-associated loci, including *RDP1*. Even though *RDP1* encodes a ribosome-biogenesis factor that would be required globally for proliferative growth, the naturally selected alleles predominantly confer reduced pollen number. This is analogous to a hypomorphic allele of the human *G6PD* gene, which encodes an enzyme in the pentose phosphate pathway and features a long haplotype because of selection for malaria resistance^24^.

The mean number of pollen grains of an outcrossing population of *Arabidopsis* *lyrata* is ~18,000 (ref. ^35^) and thus several times higher than our counts in *A. thaliana* (~2000–8000; Fig. 1e). Although the evolutionary split between the lineages leading to *A. thaliana* and *A. lyrata* is estimated to have occurred ~5 million years ago or before^36–38^, several studies suggested that predominant selfing in *A. thaliana* evolved much more recently (0–0.413 million years ago based on the timing of the loss of a self-incompatibility gene, ~0.5 million years ago based on the abundance of transposable elements, and ~0.3–1 million years ago based on the pattern of genome-wide linkage disequilibrium)^6,39,40^. Thus, in addition to presumably fixed differences to distantly related outcrossing congeners, it is quite conceivable that some underlying loci, not limited to but including *RDP1*, are still segregating within *A. thaliana*. These might reflect an ongoing selection process for the further reduction in pollen number, which has been considered a hallmark of the so-called selfing syndrome^6,8,14^. Although we note the possibility that partial sweeps of *RDP1* and other segregating loci are not directly related to the transition to predominant selfing, our analysis did not find evidence of other selective forces including local adaptation, climate association, or pleiotropic selection on other traits. Therefore, our study supports the theoretical predictions that reduced investment in male gametes is advantageous in predominantly selfing species.

Our work also illustrates that a combination of GWAS and functional analysis using a quantitative complementation test based on the CRISPR/Cas9-based alleles provides a powerful approach to dissect allelic differences underlying quantitative natural variation.

## Methods

### Pollen and ovule counting for genome-wide association studies

To perform GWAS, numbers of pollen grains and ovules per flower were counted for 144 and 151 world-wide natural accessions, respectively (Supplementary Tables 1, 2 and Supplementary Data 1). Plants were grown at 21 °C under a 16 h light/8 h dark cycle without vernalization. We grew four plants per accession. Three flower buds per plant were harvested from the main inflorescence, and each flower bud was collected into a 1.5 mL tube and dried at 65 °C overnight. We sampled individual flower buds of young main inflorescences but avoided the first and second flowers of the inflorescence because these flowers tend to show developmentally immature morphologies. We collected flower buds with mature pollen but before the anthers were opened (flower stage 13), and added 30 μL of 5% Tween 20 (Sigma-Aldrich, St. Louis, MO, USA) to each tube. The tubes were sonicated using a Bioruptor (Diagenode, Seraing, Belgium) in high power mode with 10 cycles of sonication-ON for 30 s and sonication-OFF for 30 s so that the pollen grains were released from the anther sacs. After a short centrifugation and vortexing, 10 μL of the solution was mounted on a Neubauer slide. We took three images per sample using a light microscope. The number of pollen grains per image was counted using the particle counting implemented in ImageJ (http://imagej.nih.gov/ij/) and in Fiji (http://fiji.sc/Fiji). We then estimated the total pollen number per flower based on the image size and the total volume. Ovule numbers were counted by dissecting young siliques (5.3 siliques per accession on average) under a dissecting microscope.

Because of limited chamber space, we split the plants into two batches. The two batches were treated under the same conditions in the same chambers, but at different times. We controlled for this potential batch effect for pollen number by setting equal medians and standard deviations for the two batches and used as the GWAS input of pollen number phenotype (Source Data file, Supplementary Table 1).

Sometimes, there were no or very few pollen grains per image. This was mainly in situations where anthers did not open. To eliminate these artefacts, we discarded flowers with pollen counts of <10 per image. We confirmed that such extremely low pollen numbers did not occur in specific accessions, indicating that this is not heritable.

### Plant materials and growth conditions for functional analyses

For functional analyses, we mainly used *Arabidopsis thaliana* wild-type and mutant plants of the Col-0, Bor-4, and Uod-1 accessions. The T-DNA lines SALK_064854/N666274 (*rdp1-1*) from the Salk collection^41^ and GK-879G09/N484369 (*rdp1-2*) from GABI-Kat collection^42^ were obtained from the European *Arabidopsis* Stock Centre^26^.

The T-DNA insertion in each line was confirmed using PCR with the primers listed in Supplementary Table 10 as described at http://signal.salk.edu/tdnaprimers.2.html. DNA was extracted from young leaves using the cetrimonium bromide (CTAB) method.

We generated single-nucleotide insertion/deletion lines in the Col-0, Uod-1, and Bor-4 accessions using the FAST-CRISPR-Cas9 construct (see the section CRISPR mutant) and designated them *rdp1-3* and *rdp1-4* (in Col-0), *rdp1-5* (in Uod-1), and *rdp1-6* (in Bor-4).

*Arabidopsis* seeds were sown on soil mixed with the insecticide ActaraG (Syngenta Agro, Switzerland) and stratified for 3–4 days at 4 °C in the dark. The plants were grown under 16 h of light at 22 °C, and 8 h of dark at 20 °C, with weekly treatments of insecticide (Kendo Gold, Syngenta Agro) unless noted otherwise (for GWAS, see above).

### Statistical analysis

Unless stated otherwise, statistical and population genetic analyses were performed using the statistical software R^43^. In boxplots, bars indicate the median, boxes indicate the interquartile range, and whiskers extend to the most extreme data point that is no >1.5 times the interquartile range from the box, with outliers shown by dots.

### Histological analysis of anthers

For histological analysis, inflorescences were fixed with formaldehyde:acetic acid:70% ethanol = 1:1:18 (FAA), dehydrated and embedded in Technovit 7100 according to the manufacturer’s instructions (Heraeus Kulzer GmbH, Wehrheim, Germany). Five-micrometer sections were cut with a microtome (RM2145, Leica, Germany) and stained with toluidine blue before observation under a Leica microscope (DM5000, Leica) equipped with a black-and-white camera (DFC345, Leica).

### Correlations with published GWAS results and climatic data

To examine whether pollen and ovule numbers were correlated with any of the other 107 published phenotypes^19^, or with climate and geographic variables^20^, Pearson’s correlation coefficients were calculated (Supplementary Tables 3 and 5). We also surveyed whether there were any SNPs significantly associated with climate variables in the 10-kb window including the SNP of the highest GWAS score for pollen number (Chr1:8,850,000–8,860,000). The significance was based on the genome-wide empirical *P* values^20,25^. Focusing on the same 10-kb window, we also surveyed whether there were significant SNPs (*P* < 10^–5^; minor allele frequency (MAF)>0.1) for the 107 published phenotypes in the GWAS. The correlation of pollen number and the *S*-haplogroups^21^ was also tested.

### SNP imputation

To perform GWAS, we generated a set of dense SNP markers that overlapped with the phenotyped accessions using imputation^22^. First, we constructed a reference set of 186 haplotypes from resequencing data^44,45^. MaCH version 1.0.16.c^46^ was then used to impute non-genotyped SNPs for 1311 accessions in the 250 k SNP array data set^25^. The command line used for each overlapping bin was:

mach1 --dosage --greedy -r -d [sample_bin].dat -p [sample_bin].ped -s [ref_bin].snps -h [ref_bin].haplos --prefix [output_prefix]

The output was then merged and converted into the homozygous SNP data set.

### Genome-wide association study

GWAS was performed to identify loci associated with pollen number variation in 144 natural accessions. We also performed GWAS for ovule number and for 107 published phenotypes^19^ using the same SNP set as for pollen number. The median values of pollen number were used to represent each accession. We used the log-transformed pollen number values for GWAS, as they did not deviate significantly from a normal distribution (Shapiro–Wilk normality test: *P* = 0.438). To deal with confounding effects as a result of population structure, we employed the mixed model implemented in the software Mixmogam, in which a genome-wide kinship matrix was incorporated as a random effect (population structure-corrected GWAS)^47^. By using Mixmogam, we also generated a quantile–quantile (Q–Q) plot, which shows the relationship between the observed and expected negative logarithm of *P* values (Fig. 1h). For the Manhattan plot (Fig. 1f, g), we removed SNPs with minor allele frequencies <0.15, leaving 1,115,178 SNPs overall.

### Quantitative reverse transcription PCR

For gene expression analysis, total RNA was isolated using the ZR Plant RNA MiniPrep (Zymo Research, Irvine, CA) according to the manufacturer’s instructions. Total RNA was treated with a DNA-free DNA removal kit (Thermo Fisher Scientific, Waltham, MA), and cDNA was synthesized using a High-Capacity RNA-to-cDNA kit (Thermo Fisher Scientific, Waltham, MA). For the quantitative PCR step, cDNA was used with SYBR Select Master Mix (Thermo Fisher Scientific, Waltham, MA) and gene-specific primers (Supplementary Table 10). Data were collected using the StepOnePlus Real-Time PCR System (Thermo Fisher Scientific, Waltham, MA) in accordance with the instruction manual. Expression levels were normalized using *EF1-α* (AT5G60390), which was used as an internal control.

### CRISPR/Cas9-based mutant alleles

To generate *RDP1* frameshift mutants, the CRISPR/Cas9 system was used^28^. A 20-nucleotide target sequence (5′-GCAGAAGATGAACTCCGTTC-3′) was selected using the CRISPR-P tool^48^. The target sequence was subcloned into a pTTK194 vector (pUC19 U6.26pro::sgRNA). Then, the *Hin*dIII sgRNA fragment was subcloned into a pTTK182 (pFAST-R02 35Spro::Cas9) vector or pTTK227 (pFAST-R01 RPS5Apro::Cas9). For plant transformation, the binary vector was first introduced into *Agrobacterium tumefaciens* (GV3101) and then into *A. thaliana* by the floral-dip method.

### Transgenic experiment

The *RDP1* genomic sequence was amplified by PCR using Phusion High Fidelity PCR polymerase (New England Biolabs, Beverly, MA). We used the primers 3550_At1g25260F1 (5′-TTTCTCCCCACATTTCTC-3′) and 3551_At1g25260R1 (5′-GTTTAAAATGAGAGAACCCG-3′) to amplify the full length of *RDP1* and the surrounding genomic sequence that may contain promoter and terminator regions (ca. 4.3 kbp, positions 8,857,725 to 8,853,420 on chromosome 1). The *RDP1* promoter sequence was amplified by PCR using the following primers: 3550_At1g25260F1 (5′-TTTCTCCCCACATTTCTC-3′) and 4155_At1g25260R (5′-AGCTGAGGTTTCAAATGTTGTATG-3′). These sequences were cloned into a pCR8 vector using the pCR8/GW/TOPO TA Cloning kit (Thermo Fisher Scientific, Waltham, MA). pCR8-RDP1 complete sequences were cloned into a pFAST-G01 vector^49^, and the pCR8-RDP1 promoter vector was cloned into a pGWB3 vector^50^ using Gateway LR clonase II (Thermo Fisher Scientific, Waltham, MA). We designated these constructs pFAST-RDP1 and pGWB3-RDP1pro, respectively. Plants were grown at 22 °C under a 16 h light/8 h dark cycle until transformation via *A. tumefaciens* strain GV3101 using the floral-dip method^51^. pFAST-RDP1 was transformed into *rdp1-1* homozygous mutants. Transformed seeds were selected using a fluorescence stereomicroscope with a GFP filter (Olympus SZX12, Japan). pGWB3-RDP1pro was transformed to Col-0. Seedlings carrying pGWB3-RDP1pro were selected on 1/2 Murashige and Skoog medium supplemented with 50 μg mL^−1^ kanamycin and 50 μg mL^−1^ hygromycin.

### Measuring pollen number and size with a cell counter

To expedite pollen number counting, we established a rapid method using a cell counter (CASY TT, OMNI Life Science GmbH, Germany)^52^. We found that the pollen numbers of flowers on side inflorescences and side branches of the main inflorescence were similar, but those of flowers on the main inflorescence were higher (Supplementary Fig. 18). To obtain a large number of replicates, we sampled flowers from the former two positions. We sampled during the first 3 weeks of flowering and excluded the first and second flowers on each branch; this yielded up to 40 flowers per individual. Collecting, suspending, and sonicating of flowers for GWAS were conducted as described above. All pollen solutions were suspended in 10 mL of CASYton (OMNI Life Science GmbH), and pollen numbers were counted with a CASY TT cell counter^53^. Three 400 μL aliquots of each pollen solution were counted. We counted particles within a size range of 12.5–25 μm (estimated diameter) as pollen. Samples with a clear peak at 7.5–12.5 μm were discarded as broken or unhealthy samples.

### Counting microsporocyte number

Flower buds were collected and fixed in 3:1 (vol:vol) ethanol:acetic acid for 16 h. The fixed flower buds were rehydrated in 99.5%, 70%, and 50% ethanol for 30 min each. Flower buds were transferred to 1 n NaOH for 3 h and then stained with aniline blue solution (0.1% aniline blue, 100 mm K_3_PO_4_) for 3 h. Anthers at the microsporocyte stage were dissected under a microscope with UV illumination (DM5000, Leica, Germany). Z-stack images were obtained with a confocal microscope (SP5, Leica, Germany).

### In situ hybridization

Flower buds were fixed with 3.7% formaldehyde, 5% acetic acid, 50% ethanol (FAA) and dehydrated through an ethanol series. Fixed samples were embedded in paraplast using an embedding machine (ASP200, Leica, Germany). *RDP1* cDNA was PCR-amplified using the primer pair (At1g25260g2F; 5′-tgcctaatcaaagcgagtagacc-3′ and At1g25260gR; 5′-cagagcaagttcagcttgaaagtagc-3′) and cloned into pCR4-TOPO (Thermo Fisher Scientific) vector. Cloned cDNA was used as a template for in vitro transcription using a MAXIscript T7 labeling kit (Thermo Fisher Scientific) for hybridization^54^.

### GUS assays

Plant samples were incubated in 90% acetone for 20 min at room temperature, washed with 50 mm phosphate buffer containing 0.1% Triton X-100, 2 mm potassium ferrocyanide, and 2 mm potassium ferricyanide, and incubated in the same buffer supplemented with 1 mg mL^−1^ X-Gluc for 5 h at 37 °C^55^.

### Phylogenetic analysis

Multiple sequence alignment was performed using ClustalW implemented in the CLC Workbench (version 7.7). A phylogenetic tree was generated by the neighbor-joining distance algorithm, using the aligned region (amino acid positions 44–153) and a bootstrap value of 1000. Yvh1 of *Saccharomyces cerevisiae* was used as an outgroup. Accession numbers of used sequences are listed in Supplementary Table 11.

### Selection scan

For the selection scan, we used the imputed SNP data set that was also used for GWAS. We used 298 accessions (Supplementary Data 1), covering all the accessions used for our GWAS of pollen and ovule numbers and the GWAS of 107 phenotypes reported by Atwell et al.^19,25,56^. We used the iHS statistic for the selection scan; this statistic compares the EHH between two alleles by controlling for the allele frequency of each SNP^23^. The iHS statistic uses the contrast of EHH values on each SNP; iHS values strongly deviate from zero when one allele has a long haplotype (high EHH) and the other has a short one (low EHH); the R library rehh^57^ was used to calculate the iHS statistic. The *Arabidopsis lyrata* reference genome^58^ was used to infer the ancestral state for each SNP. We first calculated the iHS for each SNP. Then, we split the genome into 10-kb windows and used the maximum score from the iHS scan for each window as the test statistic, as LD is known to decay within ~10-kb on average in geographically world-wide samples of *A. thaliana*^59^. Empirical *P* values were calculated for all windows and for all SNPs, based on their ranks in genomic distributions. To deal with the geographically biased sampling, which could be a possible confounding factor, we also performed the selection scan with 144 accessions that were used for GWAS (see Supplementary Note 2 for details; Supplementary Fig. 5).

We then asked whether the GWAS-associated windows were enriched in the extreme tails of the iHS statistic. Enrichment analysis was performed across the 10-kb windows. To examine whether enrichment of the iHS was commonly observed in GWAS peaks, we also performed this analysis for the GWAS results of the other 107 publicly available phenotypes^19^ and compared them with the GWAS of pollen and ovule numbers. Four thresholds of empirical *P* value tails were considered: 10%, 5%, 2.5%, and 1%. GWAS SNPs were considered if *P* values were smaller than 10^–4^ and the MAF was >0.1. We also performed the same enrichment analysis with MAF > 0.15 (Supplementary Fig. 4). To assess the effect of using a 10-kb window size, we also performed the iHS enrichment analysis on a per-SNP basis, in addition to the 10-kb window size (Supplementary Fig. 6).

The statistical significance of the fold-enrichment was addressed based on permutation tests that preserve the linkage disequilibrium structure in the data. A set of windows was resampled for each permutation, preserving the relative positions of the windows, but shifting them by a randomly chosen uniformly drawn number of windows for each permutation. A similar method of permutation has been used in several population genomic studies^19,25,56^. Permutation was performed 1000 times.

### Measuring plant phenotypes

For measuring rosette leaf size, we took images of plants that included a ruler at 3 weeks after germination. A minimum circumscribed circle was drawn manually on the picture using Fiji; then, the area was measured and transformed depending on the scale. The flowering date was counted as days from sowing to flowering. The dry weight of plants was measured using aerial parts, and seed weight was determined by collecting seeds from dried plants. *P* values for quantitative complementation test (Supplementary Fig. 17) are shown in Supplementary Table 12.

### Reporting summary

Further information on experimental design is available in the Nature Research Reporting Summary linked to this paper.

## Supplementary information

Supplementary information

Reporting Summary

Description of Additional Supplementary Files

Supplementary Data 1

## Data Availability

Data supporting the findings of this work are available within the paper and its Supplementary Information files. A Reporting Summary for this Article is available as a Supplementary Information file. The data sets generated and analyzed during the current study are available from the corresponding author upon request. *RDP1* gene sequence data generated in this article were registered in GenBank (National Center for Biotechnology Information) databases under the following accession numbers: LC164158 (Mz-0), LC164159 (Bor-4), LC504218 (Uod-1), LC164160 (*A. lyrata*), and LC164161 (*Arabidopsis halleri*). *P0* gene sequence data of *A. halleri* generated in this Article were registered in GenBank databases under the following accession numbers: LC164162 and LC164163. Raw and processed sequencing data for GWAS and population genetic analyses are publicly available at 10.5061/dryad.jh9w0vt7z (ref. ^60^). The source data underlying Figs. 1e, 1j, 1k, 2a, 2e, 2h, and 3, as well as Supplementary Figs. 1, 3–6, 7a, 7d, 8, 9, 10c, 10d, 15, 17, and 18 are provided as a Source Data file.

## Source data

Source Data

## Code availability

Code used for selection enrichment analysis is publicly available at https://github.com/tsuchimatsu/arabidopsis_pollen_number.

## References

[1] Harvey PH, May RM (1989). Out for the sperm count. Nature.

[2] Birkhead, T. R. & Møller, A. P. *Sperm Competition and Sexual Selection* (Academic Press, 1998).

[3] Merzenich H, Zeeb H, Blettner M (2010). Decreasing sperm quality: a global problem?. BMC Public Health.

[4] Czeizel AE, Rothman KJ (2002). Does relaxed reproductive selection explain the decline in male reproductive health? A new hypothesis. Epidemiology.

[5] Kosova G, Scott NM, Niederberger C, Prins GS, Ober C (2012). Genome-wide association study identifies candidate genes for male fertility traits in humans. Am. J. Hum. Genet..

[6] Shimizu KK, Tsuchimatsu T (2015). Evolution of selfing: recurrent patterns in molecular adaptation. Annu. Rev. Ecol. Evol. Syst..

[7] Darwin, C. *The Effects of Cross and Self Fertilisation in the Vegetable Kingdom* (John Murray, 1876).

[8] Sicard A, Lenhard M (2011). The selfing syndrome: a model for studying the genetic and evolutionary basis of morphological adaptation in plants. Ann. Bot..

[9] Barrett SCH (2002). The evolution of plant sexual diversity. Nat. Rev. Genet..

[10] Oka H-I, Morishima H (1967). Variations in the breeding systems of a wild rice, *Oryza perennis*. Evolution.

[11] Boeven PHG (2016). Genetic architecture of male floral traits required for hybrid wheat breeding. Theor. Appl. Genet..

[12] Langer SM, Longin CFH, Würschum T (2014). Phenotypic evaluation of floral and flowering traits with relevance for hybrid breeding in wheat (*Triticum aestivum L*.). Plant Breed..

[13] Goldberg EE (2010). Species selection maintains self-incompatibility. Science.

[14] Cruden RW (2000). Pollen grains: why so many?. Plant Syst. Evol..

[15] Willis JH (1999). The contribution of male-sterility mutations to inbreeding depression in *Mimulus guttatus*. Heredity.

[16] Tsuchimatsu T (2010). Evolution of self-compatibility in *Arabidopsis* by a mutation in the male specificity gene. Nature.

[17] Tsuchimatsu T, Kaiser P, Yew C-L, Bachelier JB, Shimizu KK (2012). Recent loss of self-incompatibility by degradation of the male component in allotetraploid *Arabidopsis kamchatica*. PLoS Genet..

[18] Charnov, E. L. *The Theory of Sex Allocation* (Princeton University Press, 1982).

[19] Atwell S (2010). Genome-wide association study of 107 phenotypes in *Arabidopsis thaliana* inbred lines. Nature.

[20] Hancock AM (2011). Adaptation to climate across the *Arabidopsis thaliana* genome. Science.

[21] Shimizu KK, Shimizu-Inatsugi R, Tsuchimatsu T, Purugganan MD (2008). Independent origins of self-compatibility in *Arabidopsis thaliana*. Mol. Ecol..

[22] The 1001 Genomes Consortium. (2016). 1,135 genomes reveal the global pattern of polymorphism in *Arabidopsis thaliana*. Cell.

[23] Voight BF, Kudaravalli S, Wen X, Pritchard JK (2006). A map of recent positive selection in the human genome. PLoS Biol..

[24] Tishkoff SA (2001). Haplotype diversity and linkage disequilibrium at human G6PD: recent origin of alleles that confer malarial resistance. Science.

[25] Horton MW (2012). Genome-wide patterns of genetic variation in worldwide *Arabidopsis thaliana* accessions from the RegMap panel. Nat. Genet..

[26] Scholl RL, May ST, Ware DH (2000). Seed and molecular resources for *Arabidopsis*. Plant Physiol..

[27] Jinek M (2012). A programmable dual-RNA-guided DNA endonuclease in adaptive bacterial immunity. Science.

[28] Tsutsui H, Higashiyama T (2017). pKAMA-ITACHI vectors for highly efficient CRISPR/Cas9-mediated gene knockout in *Arabidopsis thaliana*. Plant Cell Physiol..

[29] Rodríguez-Mateos M (2009). Role and dynamics of the ribosomal protein P0 and its related trans-acting factor Mrt4 during ribosome assembly in *Saccharomyces cerevisiae*. Nucleic Acids Res..

[30] Bhavsar RB, Makley LN, Tsonis PA (2010). The other lives of ribosomal proteins. Hum. Genomics.

[31] Sanders PM (1999). Anther developmental defects in *Arabidopsis thaliana* male-sterile mutants. Sex. Plant Reprod..

[32] Breuninger H, Lenhard M (2010). Control of tissue and organ growth in plants. Curr. Top. Dev. Biol..

[33] Turner TL (2014). Fine-mapping natural alleles: quantitative complementation to the rescue. Mol. Ecol..

[34] Stern DL (1998). A role of *Ultrabithorax* in morphological differences between *Drosophila* species. Nature.

[35] Willi Y (2013). Mutational meltdown in selfing *Arabidopsis lyrata*. Evolution.

[36] Koch MA, Haubold B, Mitchell-Olds T (2000). Comparative evolutionary analysis of chalcone synthase and alcohol dehydrogenase loci in *Arabidopsis*, *Arabis*, and related genera (Brassicaceae). Mol. Biol. Evol..

[37] Ossowski S (2010). The rate and molecular spectrum of spontaneous mutations in *Arabidopsis thaliana*. Science.

[38] Shimizu KK, Kudoh H, Kobayashi MJ (2011). Plant sexual reproduction during climate change: gene function *in natura* studied by ecological and evolutionary systems biology. Ann. Bot..

[39] Tang C (2007). The evolution of selfing in *Arabidopsis thaliana*. Science.

[40] de la Chaux N, Tsuchimatsu T, Shimizu KK, Wagner A (2012). The predominantly selfing plant *Arabidopsis thaliana* experienced a recent reduction in transposable element abundance compared to its outcrossing relative *Arabidopsis lyrata*. Mob. DNA.

[41] Alonso JM (2003). Genome-wide insertional mutagenesis of *Arabidopsis thaliana*. Science.

[42] Kleinboelting N, Huep G, Kloetgen A, Viehoever P, Weisshaar B (2012). GABI-Kat SimpleSearch: new features of the *Arabidopsis thaliana* T-DNA mutant database. Nucleic Acids Res.

[43] R Core Team. R Foundation for Statistical Computing (The R Foundation, 2013).

[44] Cao J (2011). Whole-genome sequencing of multiple *Arabidopsis thaliana* populations. Nat. Genet..

[45] Long Q (2013). Massive genomic variation and strong selection in *Arabidopsis thaliana* lines from Sweden. Nat. Genet..

[46] Li Y, Willer CJ, Ding J, Scheet P, Abecasis GR (2010). MaCH: using sequence and genotype data to estimate haplotypes and unobserved genotypes. Genet. Epidemiol..

[47] Segura V (2012). An efficient multi-locus mixed-model approach for genome-wide association studies in structured populations. Nat. Genet..

[48] Lei Y (2014). CRISPR-P: a web tool for synthetic single-guide RNA design of CRISPR-system in plants. Mol. Plant.

[49] Shimada TL, Shimada T, Hara-Nishimura I (2010). A rapid and non-destructive screenable marker, FAST, for identifying transformed seeds of *Arabidopsis thaliana*. Plant J..

[50] Nakagawa T (2007). Improved gateway binary vectors: high-performance vectors for creation of fusion constructs in transgenic analysis of plants. Biosci. Biotechnol. Biochem..

[51] Clough SJ, Bent AF (1998). Floral dip: a simplified method for *Agrobacterium*-mediated transformation of *Arabidopsis thaliana*. Plant J..

[52] Kakui, H., Yamazaki, M., Hamaya, N.-B. & Shimizu, K. K. in *Pollen and Pollen Tube Biology: Methods and Protocols* (ed. Geitmann, A.) (MIMB Humana Press, 2020).

[53] Tedder A (2015). Evolution of the selfing syndrome in *Arabis alpina* (Brassicaceae). PLoS ONE.

[54] Peterson KM (2013). *Arabidopsis* homeodomain-leucine zipper IV proteins promote stomatal development and ectopically induce stomata beyond the epidermis. Development.

[55] Vielle-Calzada JP, Baskar R, Grossniklaus U (2000). Delayed activation of the paternal genome during seed development. Nature.

[56] Nordborg M (2005). The pattern of polymorphism in *Arabidopsis thaliana*. PLoS Biol..

[57] Gautier M, Vitalis R (2012). rehh: an R package to detect footprints of selection in genome-wide SNP data from haplotype structure. Bioinformatics.

[58] Hu TT (2011). The *Arabidopsis lyrata* genome sequence and the basis of rapid genome size change. Nat. Genet..

[59] Kim S (2007). Recombination and linkage disequilibrium in *Arabidopsis thaliana*. Nat. Genet..

[60] Tsuchimatsu, T. et al. Adaptive reduction of male gamete number in the selfing plant *Arabidopsis thaliana*. *Dryad* 10.5061/dryad.jh9w0vt7z (2020). 10.1038/s41467-020-16679-7 PMC7280297 32514036

