# Supplementary material for: Adaptive reduction of male gamete number in the selfing plant Arabidopsis thaliana
Source: Nat Commun. 2020 Jun 8;11:2885. doi: 10.1038/s41467-020-16679-7 (PMC7280297; doi:10.1038/s41467-020-16679-7)
Supplement: Supplementary file 1 — Supplementary information [file 41467_2020_16679_MOESM1_ESM.pdf]

Adaptive reduction of male gamete number in the selfing plant *Arabidopsis thaliana*

Tsuchimatsu *et al.*

Supplementary Note 1. Correlations of pollen number with published traits and genotypes.

We analyzed the correlation of our data for pollen number per flower with the dataset of 107 published phenotypes, which includes diverse phenotypes of flowering and defense-related ionomics and developmental phenotypes, but not the numbers of ovules, flowers, or seeds. We found no significant correlations after performing Bonferroni correction for multiple testing (see main text). When multiple testing was not considered, seven traits showed a correlation ($P < 0.05$). Among these, fresh and dry weight of plants (75_FW and 76_DW, respectively, in Supplementary Table 3) were positively correlated with pollen number per flower. This suggests that larger plants produce more pollen per flower, which is consistent with empirical observations. Because plant size was measured mostly before flowering and the generation of pollen grains, these data do not suggest that larger plant size is a consequence of a larger number of pollen grains.

We also examined whether pollen number was associated with *S*-haplotypes (haplogroups A, B, or C)[1]. Because self-compatibility has been shown to have evolved independently in each haplogroup, it may be possible that different alleles are responsible for reduced pollen numbers. However, no significant correlation was found ($P = 0.682$; ANOVA; Supplementary Table 4).

Supplementary Note 2. Selection scan and enrichment analysis in various datasets and cut-offs.

To deal with possible confounding factors, we performed selection scans and iHS enrichment analyses using various datasets and cut-offs. First, we performed the same enrichment analysis with MAF > 0.15 (Supplementary Fig. 4), in addition to MAF > 0.1. When we considered MAF > 0.15, we found the significant enrichment for pollen grain number associated loci under the thresholds of top 1% and 2.5% tails (but not under 10% and 5% thresholds). Second, to deal with the geographically biased sampling, we also performed the selection scan with 144 accessions that were used for GWAS. We still found the significant iHS enrichment for pollen grain number associated loci (Supplementary Fig. 5). Third, to verify the effect of setting window, we performed the iHS enrichment analysis per SNP basis, still finding the significant iHS enrichment for pollen grain number associated loci (Supplementary Fig. 6).

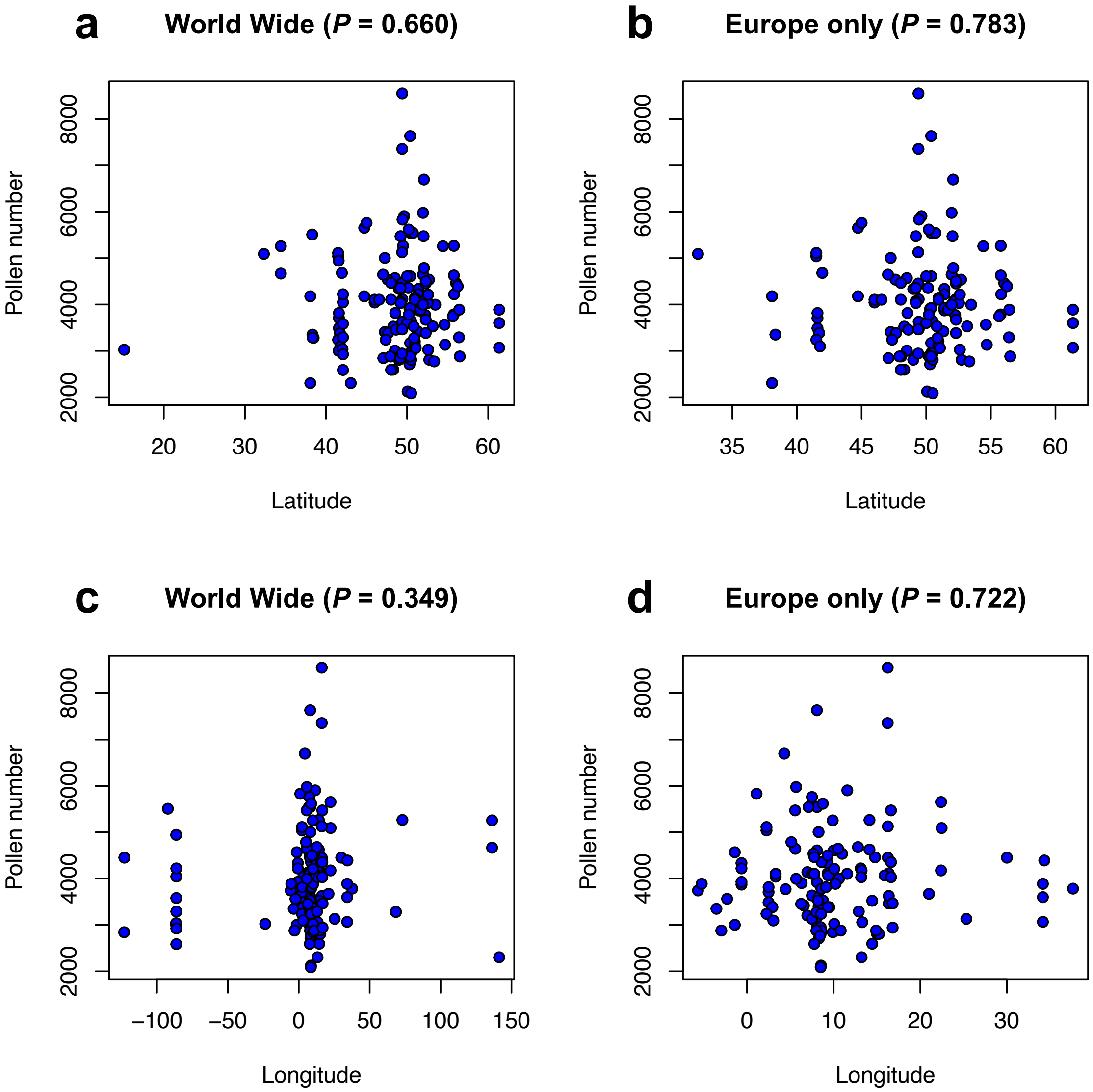

Supplementary Figure 1. Relationships between pollen number and geographic locations. (**a**) and (**b**) show the correlations between pollen number and latitude, (**c**) and (**d**) show the correlations between pollen number and longitude. (**b**) and (**d**) focus only on the European accessions. None of them showed statistically significant correlations. *P* values were calculated by two-sided tests without adjustments for multiple comparisons. Source data are provided as a Source Data file.

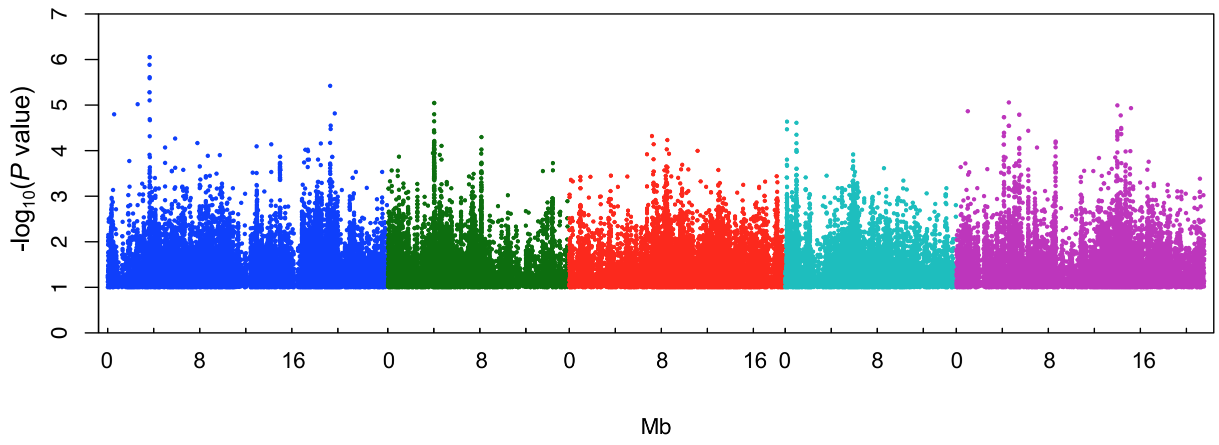

Supplementary Figure 2. Genome-wide association study of ovule number using 151 worldwide accessions.

The five chromosomes are depicted in different colors. SNPs with a minor allele frequency > 0.15 are shown. Data of ovule number is shown in Supplementary Table 2.

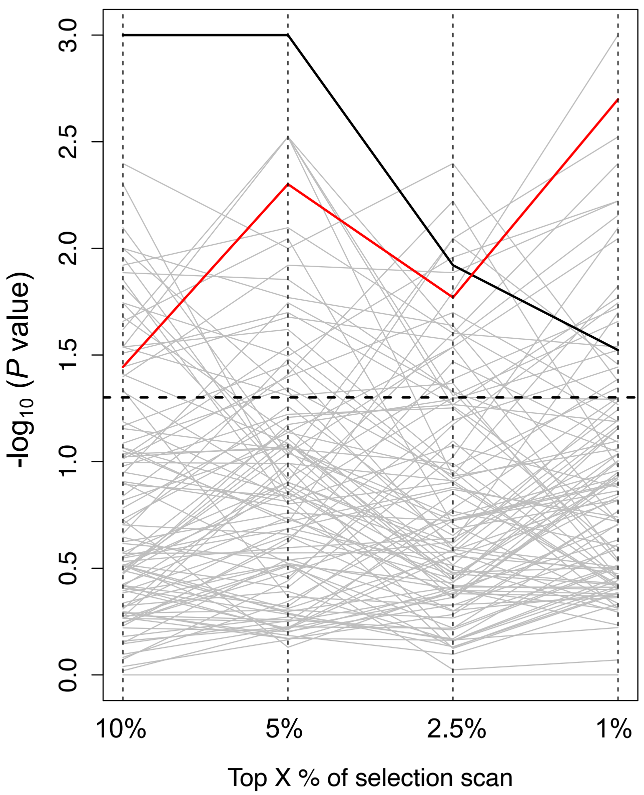

Supplementary Figure 3. *P* values of the iHS enrichment analysis based on permutation tests.

P values of the iHS enrichment analysis (Fig. 1j) are shown. Each line indicates a phenotype (red: pollen number, black: ovule number, gray: 107 phenotypes[2]). The X-axis quantifies the extreme tails of the iHS statistic. A horizontal dashed line indicates the threshold of $P = 0.05$. Note that the minimum *P* value is 0.001 because permutation was performed 1,000 times (see Methods). Source data are provided as a Source Data file.

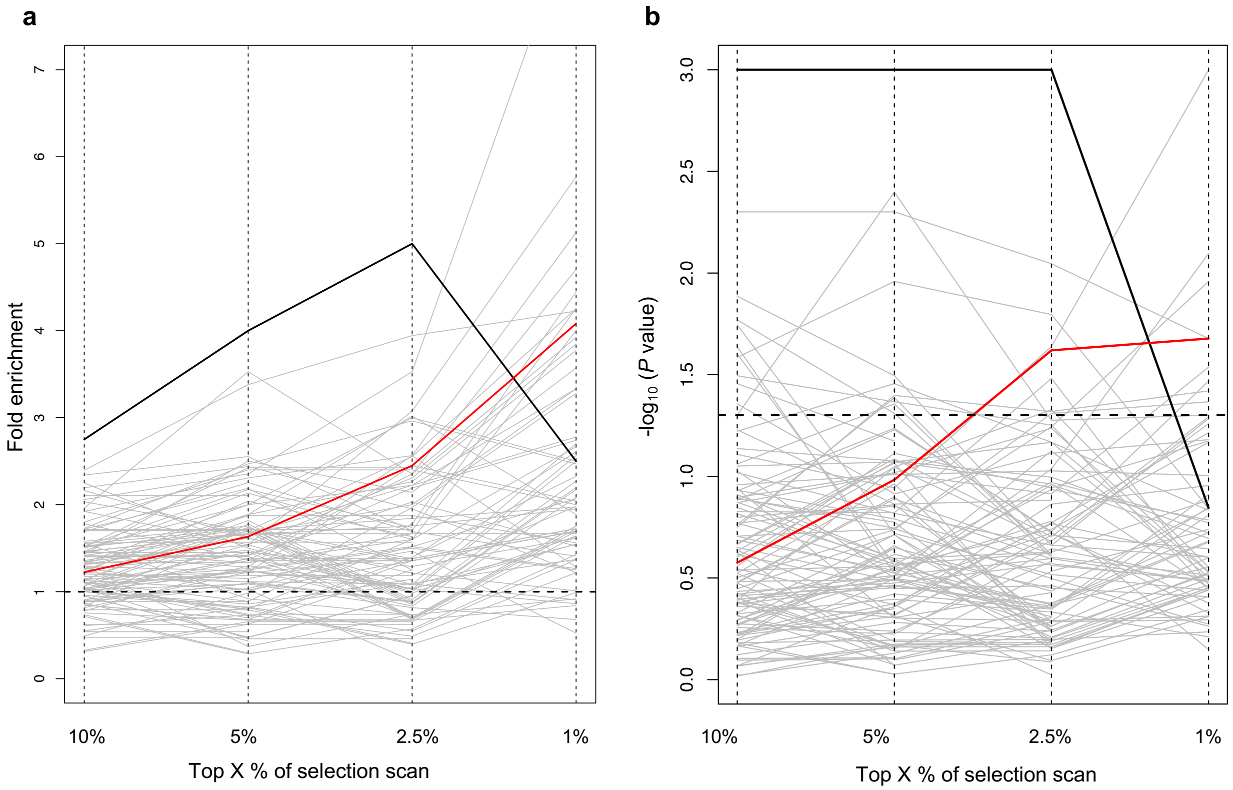

Supplementary Figure 4. Selection scan with the minor allele frequency cut-off of 0.15. Each line indicates a phenotype (red: pollen number, black: ovule number, gray: 107 phenotypes[2]). **a** The x-axis quantifies the extreme tails of the iHS statistic. **b** *P* values of the iHS enrichment analysis based on permutation tests. A horizontal dashed line indicates the threshold of $P = 0.05$. Note that the minimum *P* value is 0.001 because permutation was performed 1,000 times (see Methods). Source data are provided as a Source Data file.

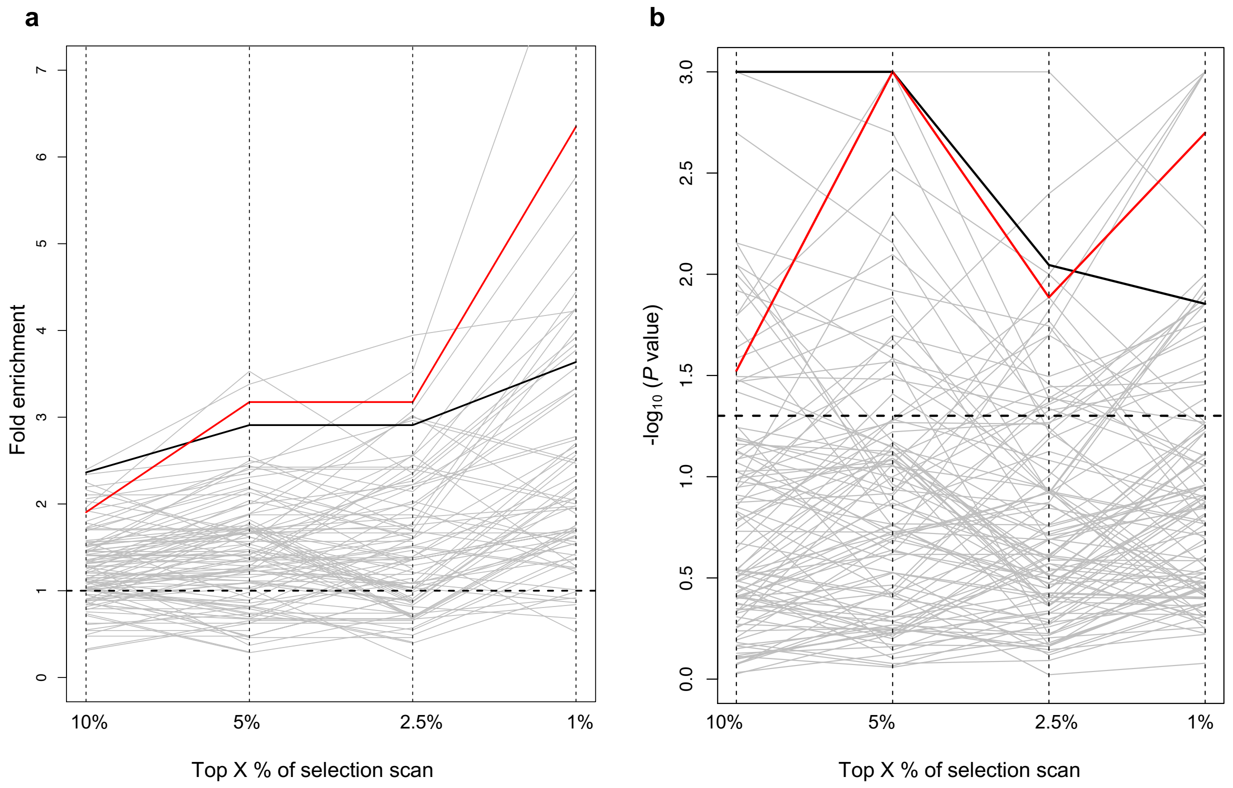

Supplementary Figure 5. Selection scan using 144 accessions that were also used for GWAS. Each line indicates a phenotype (red: pollen number, black: ovule number, gray: 107 phenotypes[2]). **a** The x-axis quantifies the extreme tails of the iHS statistic. **b** *P* values of the iHS enrichment analysis based on permutation tests. A horizontal dashed line indicates the threshold of $P = 0.05$. Note that the minimum *P* value is 0.001 because permutation was performed 1,000 times (see Methods). Source data are provided as a Source Data file.

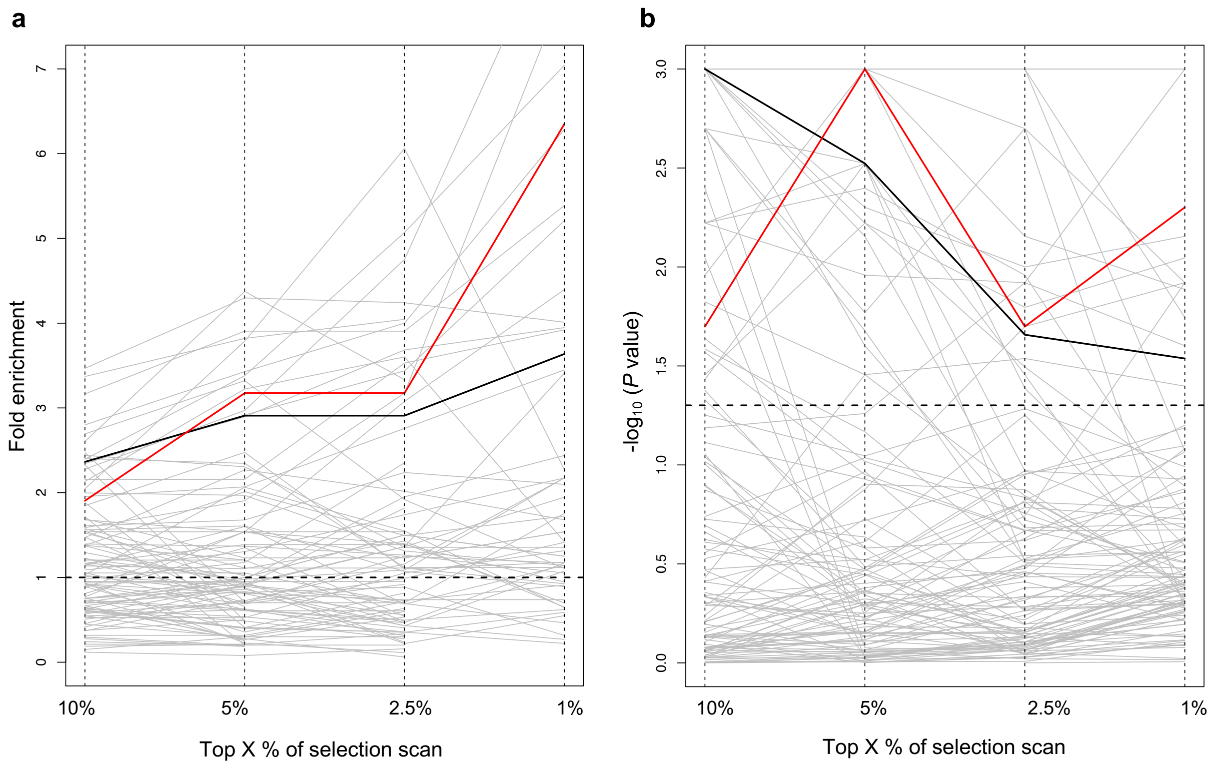

Supplementary Figure 6. Selection scan analyzed per SNP basis. Each line indicates a phenotype (red: pollen number, black: ovule number, gray: 107 phenotypes[2]). **a** The x-axis quantifies the extreme tails of the iHS statistic. **b** *P* values of the iHS enrichment analysis based on permutation tests. A horizontal dashed line indicates the threshold of $P = 0.05$. Note that the minimum *P* value is 0.001 because permutation was performed 1,000 times (see Methods). Source data are provided as a Source Data file.

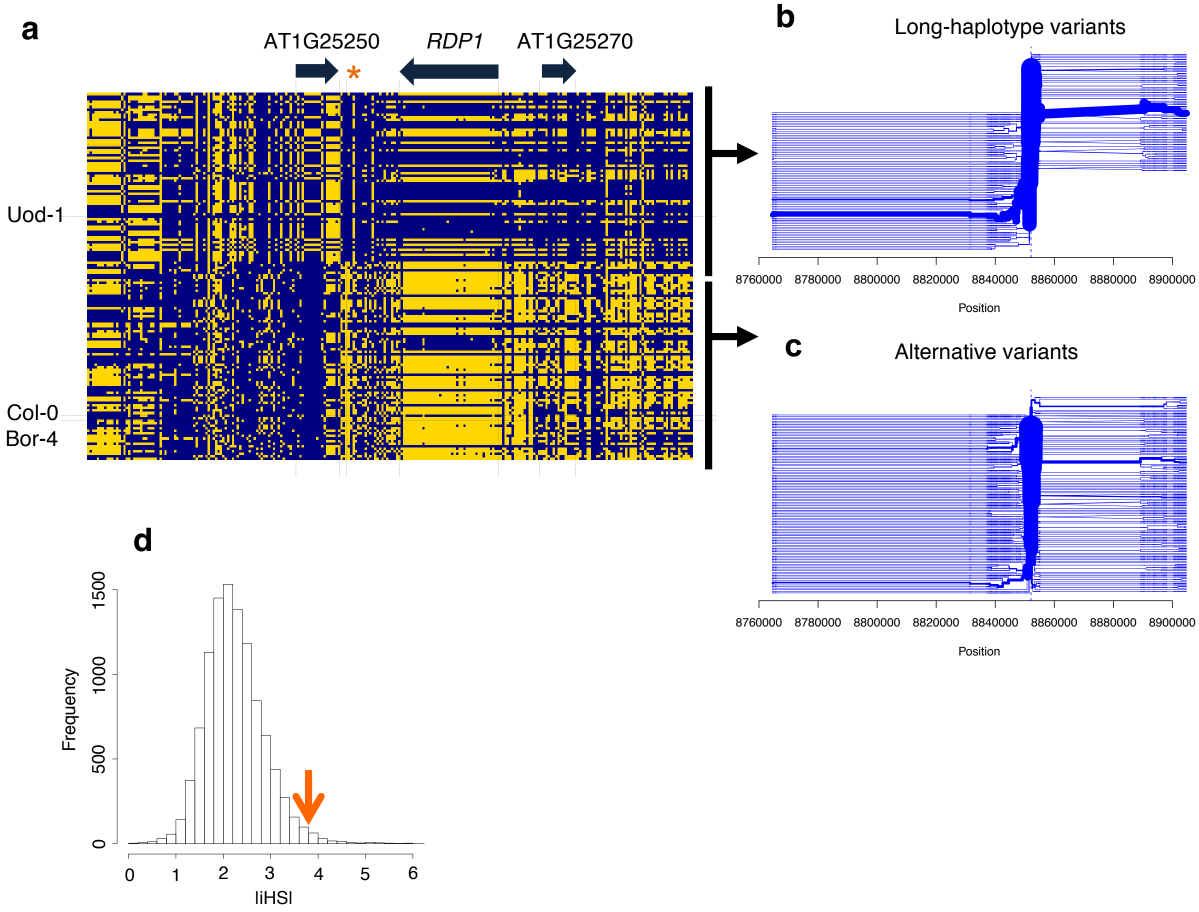

Supplementary Figure 7. A signature of selection at the *RDP1* region.
a A haplotype map of 144 accessions at the *RDP1* region. Accessions were sorted by SNP 1-8852112 (asterisk) that is significant both in the iHS scan and GWAS. Yellow and blue dots indicate polymorphisms. **b, c** Haplotype bifurcation diagrams for the long haplotype (**b**) and the other variants (**c**) in the *RDP1* region. Line width corresponds to the number of individuals with the indicated haplotype. **d** Genome-wide distribution of the absolute values of the iHS statistics. The *RDP1* region is in the upper tail of the distribution (arrow, empirical $P = 0.0149$, one-sided test). Source data underlying Supplementary Fig. 7a and 7d are provided as a Source Data file.

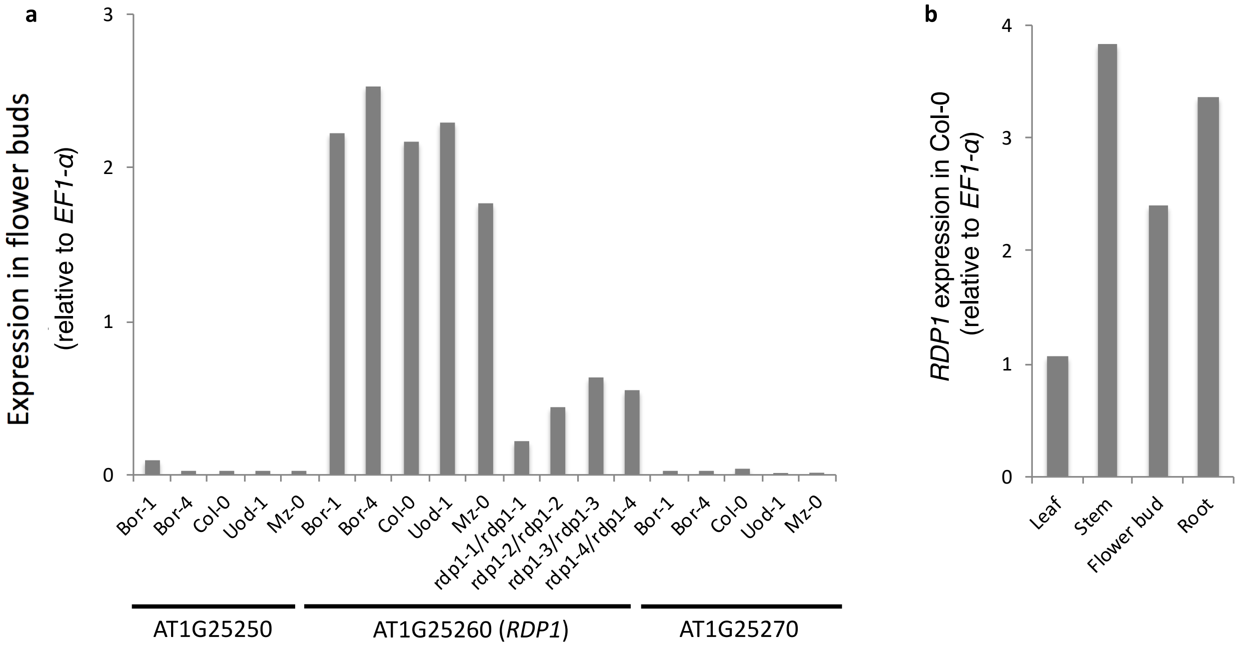

Supplementary Figure 8. Expression levels of *RDP1* and neighboring genes.
a Expression of *RDP1* (AT1G25260) and neighboring genes (AT1G25250 and AT1G25270) in flower buds of natural accessions and mutants. **b** Expression levels of *RDP1* in leaf, stem, flower bud, and root of the Col-0 accession. Gene expression levels were normalized to *EF1-α* (AT5G60390). Source data are provided as a Source Data file.

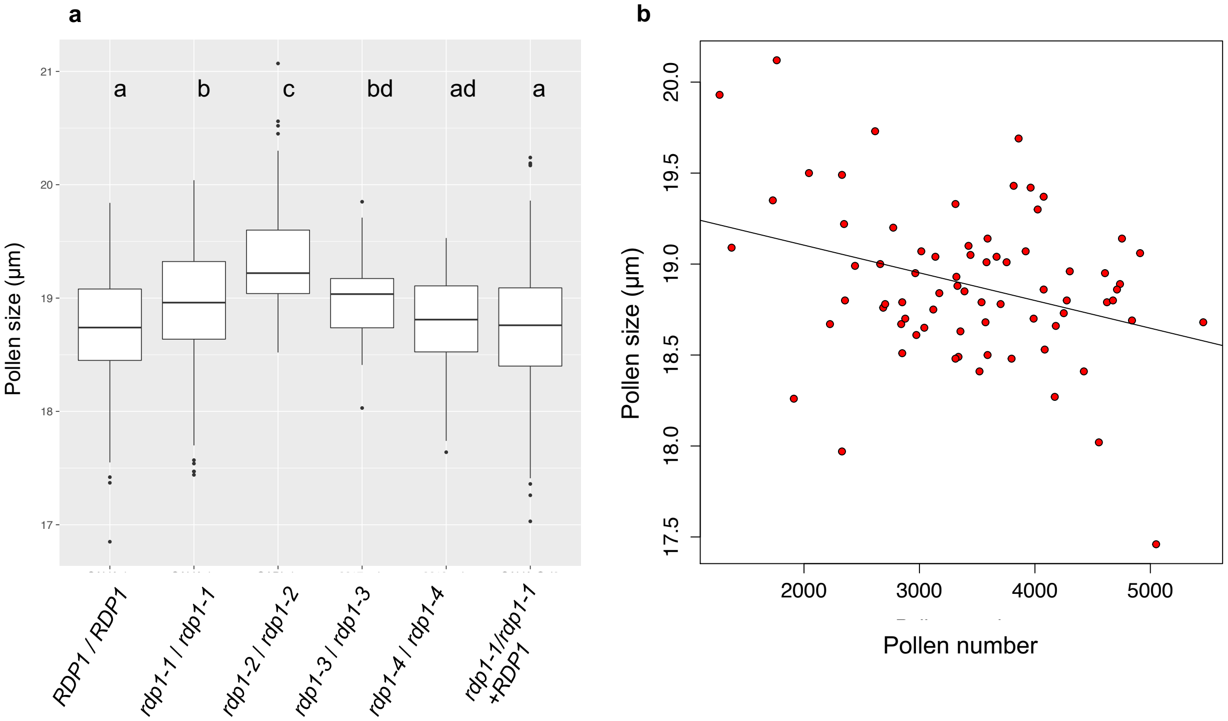

Supplementary Figure 9. Pollen size in *RDP1* mutants and its correlation with pollen number.
a Pollen size differences between four types of homozygous mutants (wild type (*RDP1*/*RDP1*), *rdp1-1/rdp1-1, rdp1-2/rdp1-2*, *rdp1-3/rdp1-3*, and *rdp1-4/rdp1-4*), and the lines complemented with the Col-0 allele (*rdp1-1*/*rdp1-1*+*RDP1*). Letters (a–d) indicate significant differences in pollen number or size between lines, determined by a nested analysis of variance and *post hoc* Tukey test ($P < 0.05$; See Supplementary Table 9 for P values of each comparison). Numbers of flowers measured (left to right, as plotted): N = 135, 132, 45, 40, 60, 411. Boxplots show center line: median; box limits: upper and lower quartiles; whiskers: not greater than 1.5 times the interquartile range; point: outlier. **b** Pollen size is negatively correlated with pollen number in the Col-0 accession (N = 73, $P = 0.00501$). This negative correlation may be attributed to developmental constraints[3]. Source data are provided as a Source Data file.

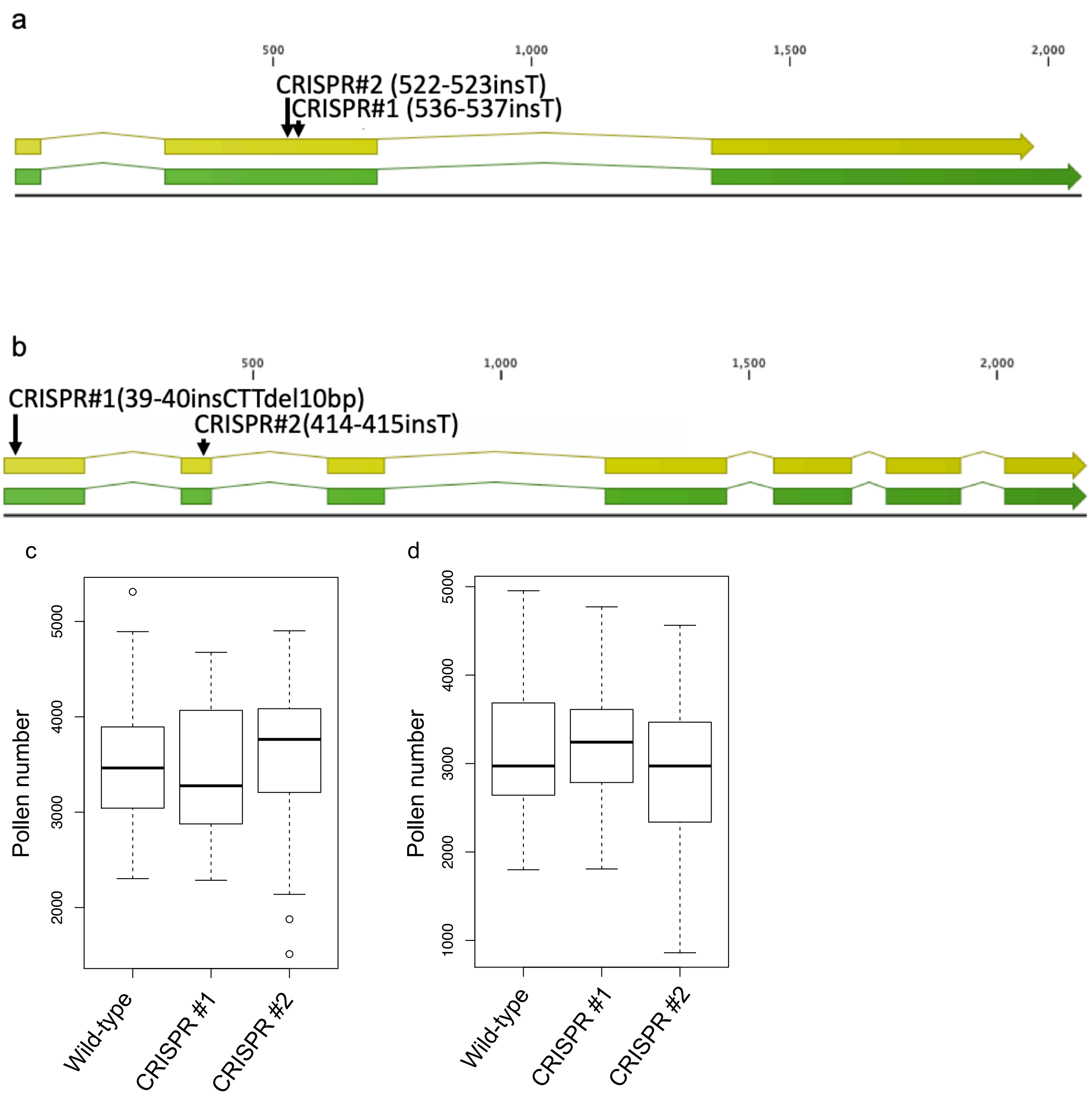

Supplementary Figure 10. Function of neighboring genes of *RDP1*.
a, **b** Gene structures of AT1G25250 (**a**) and AT1G25270 (**b**). Yellow and green boxes indicate coding region and exon respectively. Numbers indicate base pair from start codon. Each mutation points are shown by arrows. **c, d** Pollen number did not differ significantly between wild type and the null mutants by CRISPR-Cas9 (**c**; AT1G25250, **d**; AT1G25270). Two-sided *t*-test against wild type: CRISPR #1 of AT1G25250, $P = 0.5757$; CRISPR#2 of AT1G25250, $P = 0.4115$; CRISPR#1 of AT1G25270, $P = 0.4224$; CRISPR#2 of AT1G25270, $P = 0.2275$. Boxplots show center line: median; box limits: upper and lower quartiles; whiskers: not greater than 1.5 times the interquartile range; point: outlier. Number of flowers pollen-counted (left to right, as plotted): N = 46, 18 and 54 for (**c**); N = 54, 127 and 57 for (**d**). Source data underlying Supplementary Fig. 10c and 10d are provided as a Source Data file.

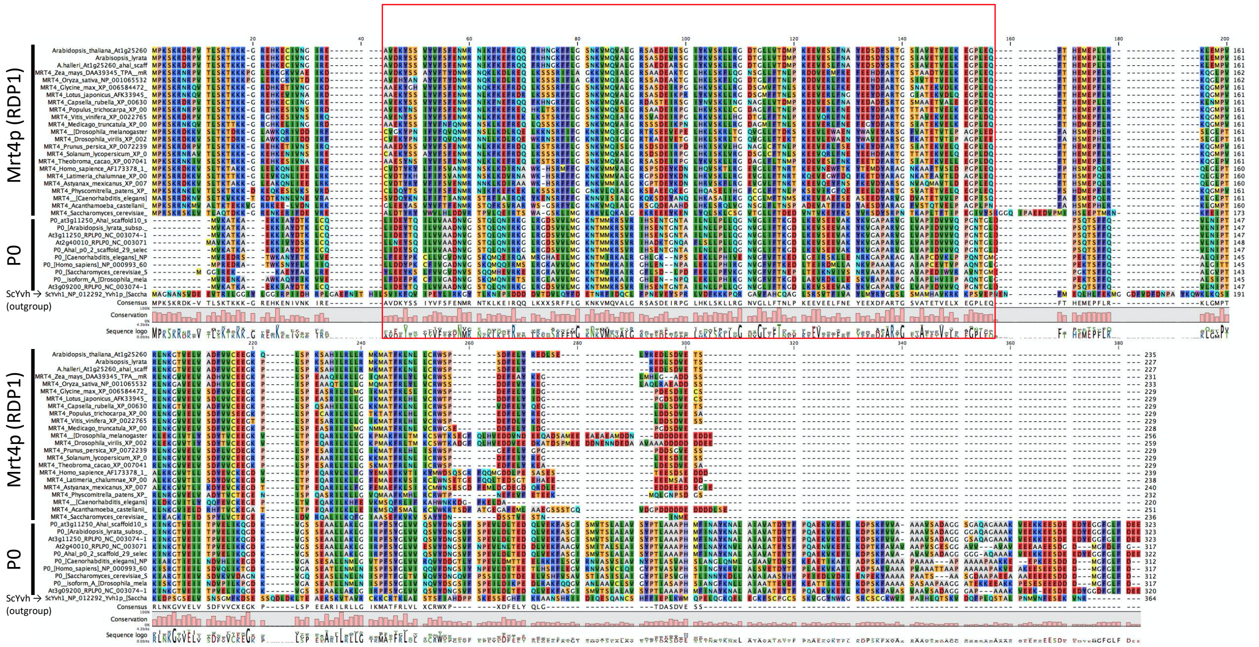

Supplementary Figure 11. Alignment of RDP1/Mrt4p and P0 protein sequences generated by ClustalW.

Accession numbers of used sequences are listed in the Supplementary Table 11. The phylogenetic tree (Supplementary Fig. 12) was generated using the aligned region indicated by the red box (44–153 aa).

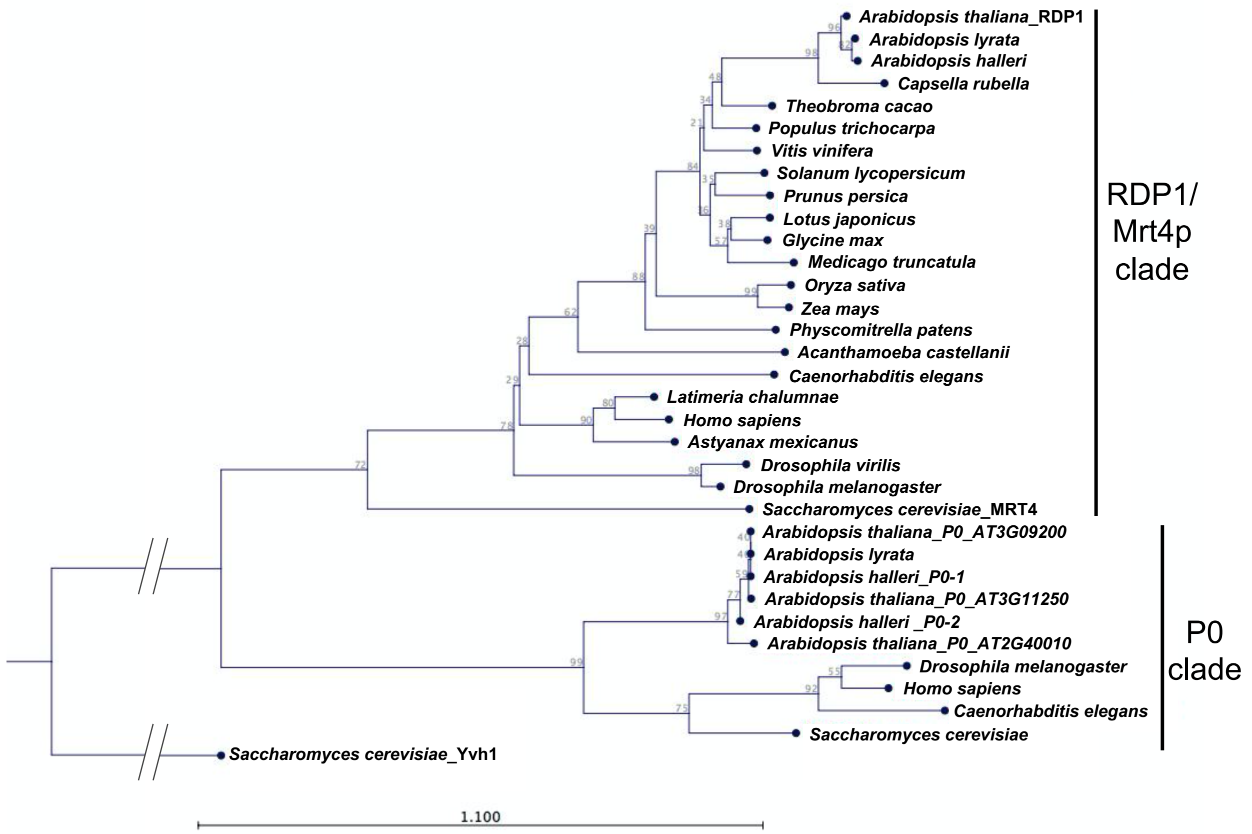

Supplementary Figure 12. Phylogenetic tree of RDP1/Mrt4p and P0.
Phylogenetic tree was constructed by using the neighbor-joining method[4]. The scale bar indicates the genetic distance based on the Kimura model[5]. Values on the branches indicate the percentage of 1,000 bootstrap replicates. See Supplementary Fig. 11 for the alignment used for this tree. ScYvh1 was used as an outgroup.

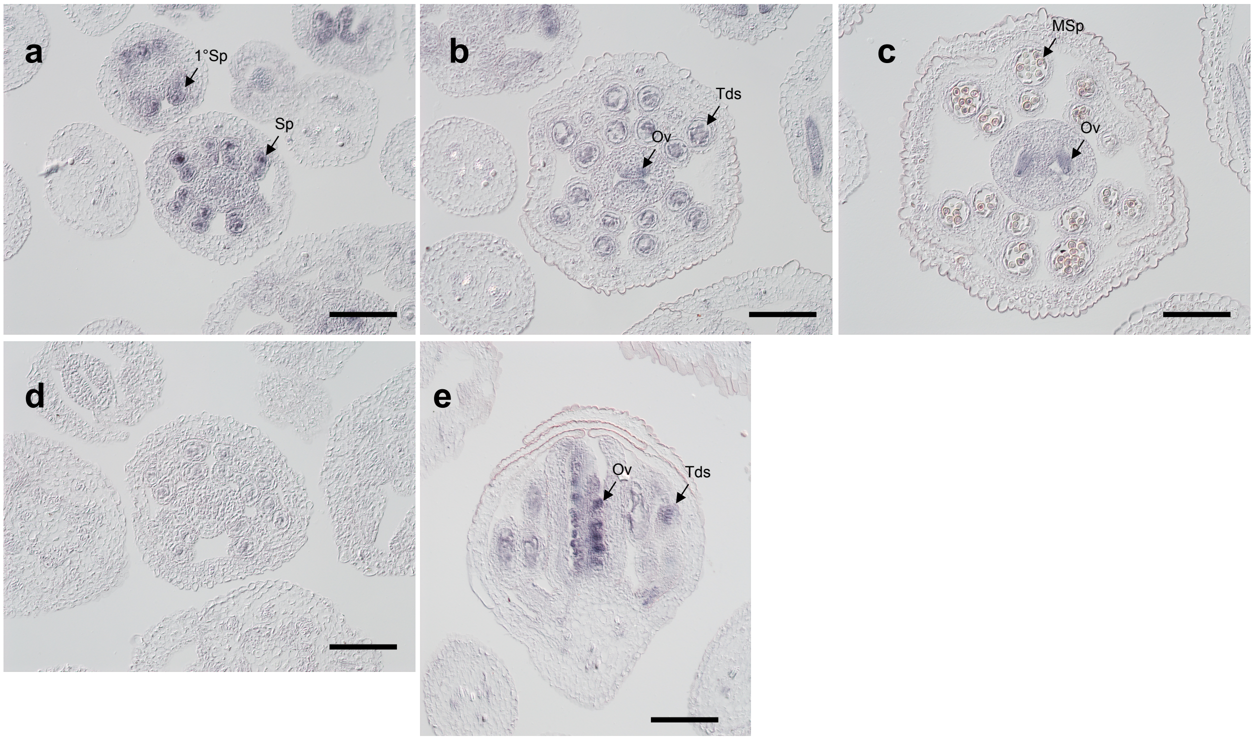

Supplementary Figure 13. *In situ* hybridization analysis of *RDP1*.
RDP1 expression was analyzed in Col-0 with *RDP1* antisense (**a**, **b**, **c**, and **e**) or sense (**d**) probes. Transverse (**a**–**d**) and longitudinal (**e**) cross sections of the flower buds. *RDP1* expression was detected at the early stages of pollen lineage such as primary sporogenous layer (1°Sp in stage 7 flowers in (**a**)), sporogenous cell (Sp in stage 8 flowers in (**b**)), and tetrad (Tds in stage 10 flowers (**b**, **e**)), but not in the microspore (MSp in stage 11 flowers in (**c**)). *RDP1* expression was also detected in stage 10 and 11 ovules (Ov in (**b**, **c**, **e**)). Scale bars; 100 μm. A similar expression pattern was observed at least two individuals (**a**-**e**).

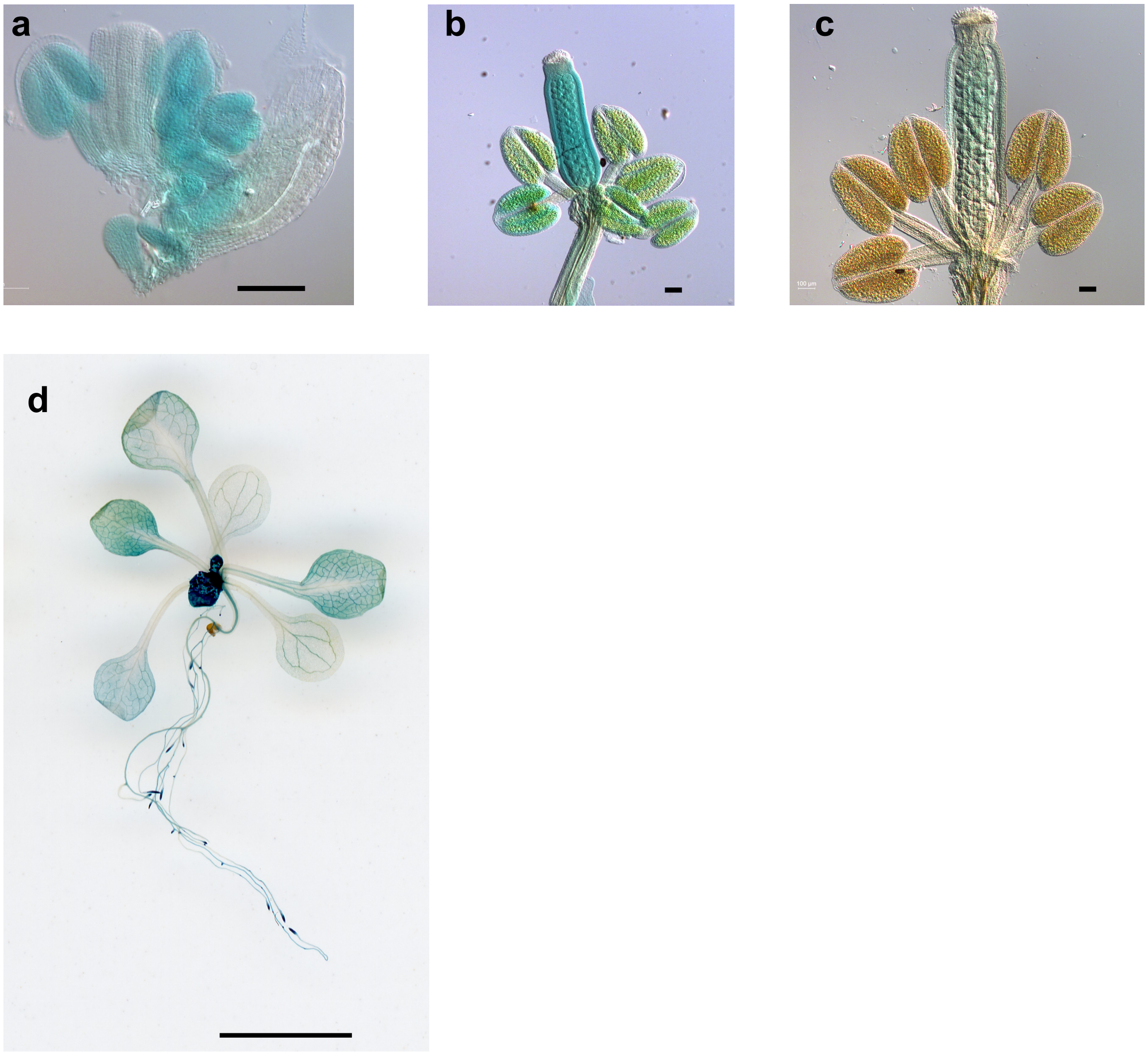

Supplementary Figure 14. GUS staining patterns of plants with *RDP1pro::GUS* construct. GUS-stained samples were observed at flower stage 9 (**a**), 11 (**b**), 13 (**c**), and seedling (**d**). Scale bars: 100 µm for **a**–**c**, 5 mm for **d**. *RDP1* expression was preferentially detected in young stamens (**a**), in pistils (**b**), and in shoot and root apical meristems and young leaves (**d**). A similar GUS staining pattern was observed at least four individuals (**a**-**d**).

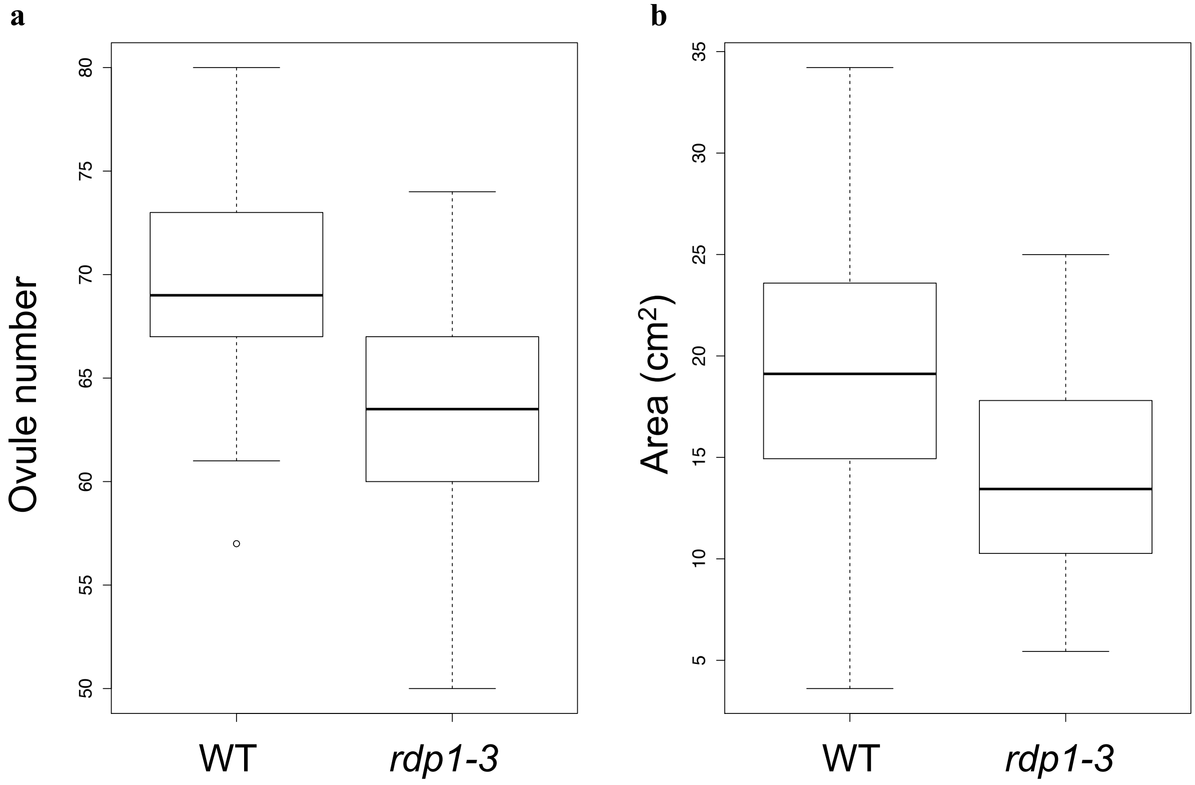

Supplementary Figure 15. Pleiotropy of *rdp1-3*.
a Ovule number per flower was reduced in *rdp1-3* (N = 42, wild type; N = 53, *rdp1-3/rdp1-3*; *t* test, $P = 2.34 \times 10^{-8}$). **b** Rosette area was smaller in *rdp1-3* (N = 49, wild type; N = 27, *rdp1-3/rdp1-3*; two-sided *t* test, $P = 0.000986$). Boxplots show center line: median; box limits: upper and lower quartiles; whiskers: not greater than 1.5 times the interquartile range; point: outlier. Source data are provided as a Source Data file.

a

```
Col-0 GCCATATAGC ACGACATATA TATAGCCCAC ATACAATCAA TATTGCTCCT AGTAAAAATA AAAATAAATA TATTGCTTCA AGTACACTTC AATTTGAAAA 100
Uod-1 .......... .......... .......... .......... .......... ........G. .......... .......C.. ...G...... .......... 100
Bor-4 .......... .......... .......... .......... .......... .......... .......... .......C.. .......... .......... 100

Col-0 CAATAATATA GTGTATGCTT ATAAATATTA TCAAATCTTA ATAAATAAAT TGTCTTTTAC CTTCCCAAAT AAACTTCTCG TCCAAGAACA AAACACAAGC 200
Uod-1 .......... .......... .......... .......... .......... .......... .......... .......... .......... .......... 200
Bor-4 .......... .......... .......... .......... .......... .......... .......... .......... .......... .......... 200

Col-0 CACGGCGACT ACTACGAGCA TGAGAAGGGT TGAGGACTTC TACAAATGAG TGGACCCATC CCCTTAGTCT CGAACAAATT GCCAGTAGTG AGATCACTAC 300
Uod-1 ....A..... .......... .......... .......... ......A... .........G .......... .......... .......... .......... 300
Bor-4 .......... .......... .......... .......... ......A... .......... .......... .......... .......... .......... 300

Col-0 TCACTACTAA TGGTTGTTCC TATTGTTCCC TATACGTTTA CATTGTTTAA TTTCCGAGCA TTCATATAAA TAATTTATAA TTCATTATTA AGAGGTTTAA 400
Uod-1 .......... ...C...... C......... .......... .......... .......... .......... .......... ..T....... .......... 400
Bor-4 .......... .......... C......... .......... .......... .......... .......... .......... .......... .......... 400

Col-0 TAATATGATT TTGAAAGTAC CACTTTTTAA TTAATTTTTA TAACATTAAA TTTAAAACTT TAATTTCTTA ACTTAAAAAC TTTTGCACAA CAAAACAAAA 500
Uod-1 .......... .......... .......... .......... .......... .......... .......... .......... .......... .......... 500
Bor-4 .......... .......... .......... .......... .......... .......... .......... .......... .......... .......... 500

Col-0 AAAACTAAAA ACACTATAAT CCATTTAAAT TTAAACTAGT ACACCCTCCA ATGACAAAAA AAAATCGGCC CAGATTTCTG ATAGCTAAAA GGCCTGAAAC 600
Uod-1 .......... .......... .......... .......... .......... .......... .......... .......... .......... .......... 600
Bor-4 .......... .......... .......... .......... .......... .......... .......... .......... .......... ........T. 600

Col-0 GGGCCGGATC CGGATCGGGT TGAATTAGGG GTCCAGATTA AAAAGTGCGA ATTAAAGCAA ACAAGGGAAC AAGGGTTTCA GTTTCCCTGA AGAAAATTGA 700
Uod-1 .......... .......... .......... .......... .......... .......... .......... G......... .......... .......... 700
Bor-4 .......... .......... .......... .......... .......... .......... .......... G......... .......... .......... 700

Col-0 AAGGAGCTTA ATAAACAGAA GCCGTTCTCC TTCACTTTCG TTCCTCTCAC TTCCATACAA CATTTGAAAC CTCAGCTATG CCTAAATCAA AGCGAGATAG 800
Uod-1 .......... .......... .......... .......... .......... .......... .......... .......... .......... .......... 800
Bor-4 .......... .......... .......... .......... .......... .......... .......... .......... .......... .......... 800

Col-0 ACCAGGTTAG AACTCTTCTT CCTTCACTCT TTCTGCTCTC TATTGTCTGA ACCATTGTAT CTCTGTCACT GGTTTTGGAA GCTGTAACTT TTGTAGCCGC 900
Uod-1 .......... .......... .......... .......... .......... .......... .......... .......... .......... .......... 900
Bor-4 .......... .......... .......... G........G .......... .......... .......... .......... .......... .......... 900

Col-0 CGTCAAGGGT TTTAGGGTTT TGTTAGGTTT TTAGAGGAAC CCTTTTGAAA AAAACTTTAA CTTGTTGGAG TTAGTAATGT TTTTTCATAG CTTCTATGGT 1000
Uod-1 .......... .......... .......... .......... .......G.. .......... .......... .......... .......... .......... 1000
Bor-4 .......... .......... .......... .......... .......... .......... .......... .......... .......... .......... 1000

Col-0 TGAATCGATT GATGAATGGT TTATCTAAGG GTTTTGTTTT CTGATTTTTT GCTGCAGTGA CTTTGTCTAA GACGAAGAAG AAAGGAAGAG AGCATAAGGA 1100
Uod-1 .......... .......... .......... .......... .......... .......... .......... .......... .......... .......... 1100
Bor-4 .....T.... .......... .......... .......... .......... .......... .......... .......... .......... .......... 1100

Col-0 GTGTATTGTG AATGGAATTA GGGAGGCTGT TGAAAAATAT TCCTCTGTCT ATGTTTTCTC TTTTGAAAAC ATGAGAAATA TCAAATTCAA GGAGTTTAGG 1200
Uod-1 .......... .......... .......... .......... .......... .......... .......... .......... .......... .......... 1200
Bor-4 .......... .......... .......... .......... .......... .......... .......... .......... .......... .......... 1200

Col-0 CAGCAGTTTA GGCACAATGG AAAGTTAGTT TCTTTGTCCT TCTTCTGGTT GATGTTAGAA TTACTTGAAT GTTATGACTG ACTCGGTTCT TATTTGTCTA 1300
Uod-1 .......... .......... .......... .......... .......... .......... .......... .......... .......... .......... 1300
Bor-4 .......... .......... .......... .......... .......... .......... .......... .......... .T........ .......... 1300

Col-0 GGTTCTTCCT AGGTTCGAAC AAAGTGATGC AGGTTGCTCT TGGTCGTTCT GCAGAAGATG AACTCCGTTC TGGTATCTAC AAAGTCTCCA AGGTTTGTGT 1400
Uod-1 .......... .......... .......... .......... .......... .......... .......... .......... .......... .......... 1400
Bor-4 .......... .......... .......... .......... .......... .......... .......... .......... .......... .......... 1400

Col-0 ATTCTGCTTC TTACAATGGT TCTTTTATGT TAAATGGTCA TTTTTTGTCA GTTAGATTTA CATATGTTGT GGAATGTTGT TTCAGCTGCT TCGTGGTGAT 1500
Uod-1 .......... .......... .......... .......... .......... .......... .......... .......... .......... .......... 1500
Bor-4 .C........ .......... .......... ....C..G.T ..C......T .......... ....C..... .......... .......... .......... 1500

Col-0 ACTGGACTTC TTGTTACTGA TATGCCAAAG GAAGAAGTCG AAAGGTATCT ATCTATGTAT CTCTTGACGT CCTCTTTTTT CCTCTTTTGC TTTGGTGAAA 1600
Uod-1 .......... .......... .......... .......... .......... .......... .......... .......... .......... .......... 1600
Bor-4 .......... .......A.. .......... ........T. .......... ...CT.---- .......AA. ..CTC..... A..G.GG..- ------A... 1589

Col-0 CAACAGTGAC GGGTTTGAAT TGTATACATG TCTAGTTGTT GTAACTGTTC ACAATACTTC TTCTGGTTGT GTATTGCAGC TTATTTAACG CTTATGAGGA 1700
Uod-1 .......... .......... .......... .......... .......... .......... .......... .......... .......... .......... 1700
Bor-4 .......A.G T......... .....C..A. ...CT...G. .......C.. .....GA... .........C --........ .......... .......... 1687
```

```
Col-0 TTCCGACTTT TCAAGGACCG GAAGCATTGC AGTAGAAACG GTTTGTGAAC AGAACTTGTG CCATCTCTAT ATACTGTTGT TTCTAGATAA ATAAATAAGC 1800
Uod-1 .......... .......... .......... .......... .......... .......... .......... .......... .......... .......... 1800
Bor-4 .......... .......... .......... .......... .........T G......... .......... ..C....... .......... .......... 1787

Col-0 TTGACTATGA TCAGGCTCTG CT--TCTCTT TGTTATTTAA AAAACTTTGT TTCGTTACTT TTATCTTAAT TACTATATTG GTACCACCTA AATGATTAGG 1898
Uod-1 .......... .......... ..CT...... .......... .......... .......... .......... .......... .......... ......A... 1900
Bor-4 ....G..... .......... ..--...... .......CC. .......... .......... .......... ....C.G... .........G T......... 1885

Col-0 TTGAACTGAA GGAAGGTCCT CTAGAACAGT TCACACATGA GATGGAACCA CTTCTTCGGA AGCTTGAAAT GCCTGTACGA CTAAACAAAG GTCTGTCGTC 1998
Uod-1 .......... .......... .......... .......... .......... .......... .......... .......... .......... .......... 2000
Bor-4 .......... .......... .......... .......... .......... .......... ....G..G.. ......T... .......... ..T.....C. 1985

Col-0 TCACCATCTT AATATTCCTC TTTCTCATAC TCACTTTTGC TATCTATATT AATCCAAAAA CTTATTTCTT CTTCTTATTT TAATATGTAG GTACTGTGGA 2098
Uod-1 .......... .......... .......... .......... .......... .......... .......... ..G....... .......... .......... 2100
Bor-4 .......... ....A..... --------.T .......... .........A .......... .......... .......... .......... .......... 2077

Col-0 GTTGGTCGCT GACTTTGTAG TTTGTGAAGA AGGAAAACAA CTTTCGCCAA AATCAGCCCA CATTTTGGTA ATAAGACTTC TGTATACCAA AACCCTTTCC 2198
Uod-1 .......... .......... .......... .......... .......... .......... .......... .......... .......... .......... 2200
Bor-4 .......... .......... .......... .......... .......... .......... .......... .......... .......... .......... 2177

Col-0 TTTTAATTTG TATGCTTTAT TTCTTATACT TGTTTGTTTA CTTTGCAGCG TCTATTGCGA ATGAAGATGG CTACTTTCAA GCTGAACTTG CTCTGCAGAT 2298
Uod-1 .......... .......... .......... .......... .......... .......... .......... .......... .......... .......... 2300
Bor-4 .......... .......... .......... .......... .......... .......... .......... .......... .......... .......... 2277

Col-0 GGAGCCCTAG TGACTTTGAG CTTTACAGAG AAGATCTGTC AGAGCTTTAC CGAGAAGATC TCTCAGACGT TGAAACCTCT TAGGAAGCCA AGATTTTGAC 2398
Uod-1 .......... .......... .......... .......... .......... .......... .......... .......... .......... .......... 2400
Bor-4 .......... .......... .......... .........T .......... .......... .......... .......... .......... .......... 2377

Col-0 TCCACCCTTT AAATTTTTCT TAATATCATC ACTATCACAT GGATTTTATA TTGTGGCACA TGGTTTGCAT TCAAATGTTT TAAGTCAAAG TTTTGCTTGC 2498
Uod-1 .......... .......... .......... .......... .......... .......... .......... ..G....... .......... .......... 2500
Bor-4 .......... .......... .......... .......... .......... .......... .......... ..G....... .......... .......... 2477

Col-0 TTTACATGTT TTGTAGAATA TCAACTTATC TTCAAGAACC CCCTATCCTA ATATTGGCAA ATCAATCTTG AAAACCTTGT TTTTCTTATT CAGCCACATT 2598
Uod-1 .......... .......... .......... .......... .......... .......... .......... .......... .......... .......... 2600
Bor-4 .......... .......... .......... .......... .......... .......... .......... .......... .......... .......... 2577

Col-0 ATGGAATTTC TTACTCAATT TGCTTACTTT TTACAGATTT ATAGATGGTG TTGGGACCGA ATTAATTGGC ATGCGTATGA TAATTTAAAG CAAGTTTTTC 2698
Uod-1 .......... .......... .......... .......... .......... .......... .......... .......... .......... .......... 2700
Bor-4 .......... .......... .......... .......... .......... .......... .......... .......... .......... .......... 2677

Col-0 TTCTTGTCTT TAAAATTATT GTGGTCCATG GGCTTGAGAG CAGTCCAACA ACGTTCACAC GTCGTCCATC TCTTTTGATA ATTTCTTGTT T-TTTGTTTT 2797
Uod-1 .......... .......... .......... .......... .......... .......... .......... .......... .......... .G...T.... 2800
Bor-4 ....C..... .......... .......... .......... .......... .......... .......... .......... .......... .G...T.... 2777

Col-0 TTTGTTTGTT TTTTAGCAAT CCGAAACTAT TGCGTTTGAC TAGGTGAGCT TAGAGTTGTG ATGTTAGGGA TAGAAGCAGT TTTCTACGAA AGGGGTTGAA 2897
Uod-1 .......T.C .......... .......... .......... .......... .......... .......... .......... .......... ......-... 2899
Bor-4 .......T.C .......... .......... .......... .......... .......... .......... .......... .......... .......... 2877

Col-0 GATACTATGA GGCGAATGCG GTTGAACAAA ATAGAACCCA CCGGAGATTT TGGTAAAAGA TTAAACGGAA AGGCGATACT TCCAACTCCG ACGTAACAAA 2997
Uod-1 .......... .......... .......... ..C....... .......... .......... .......... .......... .......... .......... 2999
Bor-4 .......... .......... .......... .......... .......... .......... .......... .......... .......... .......... 2977

Col-0 TGTTTAGTTG TAGATTTTCA ACATTTT 3024
Uod-1 .......... ...G...... ....... 3026
Bor-4 .......... ...G...... ....... 3004
```

b

```
Col-0 MPKSKRDRPV TLSKTKKKGR EHKECIVNGI REAVEKYSSV YVFSFENMRN IKFKEFRQQF RHNGKFFLGS NKVMQVALGR SAEDELRSGI YKVSKLLRGD 100
Uod-1 .......... .......... .......... .......... .......... .......... .......... .......... .......... .......... 100
Bor-4 .......... .......... .......... .......... .......... .......... .......... .......... .......... .......... 100
Col-0 TGLLVTDMPK EEVESLFNAY EDSDFSRTGS IAVETVELKE GPLEQFTHEM EPLLRKLEMP VRLNKGTVEL VADFVVCEEG KQLSPKSAHI LRLLRMKMAT 200
Uod-1 .......... .......... .......... .......... .......... .......... .......... .......... .......... .......... 200
Bor-4 .......... .......... .......... .......... .......... .......... .......... .......... .......... .......... 200
Col-0 FKLNLLCRWS PSDFELYRED LSELYREDLS DVETS* 236
Uod-1 .......... .......... .......... ...... 236
Bor-4 .......... .......... .L........ ...... 236
```

Supplementary Figure 16. Sequences of the *RDP1* gene.

Nucleic acid (**a**) and amino acid (**b**) sequences of Col-0, Uod-1, and Bor-4 are shown. **a** Nucleic acid sequences containing *RDP1* with 777 bp upstream and 643 bp downstream sequences. **b** Amino acid sequences of RDP1. Dots represent identical sequence with Col-0. Dashes indicate deletion sequence.

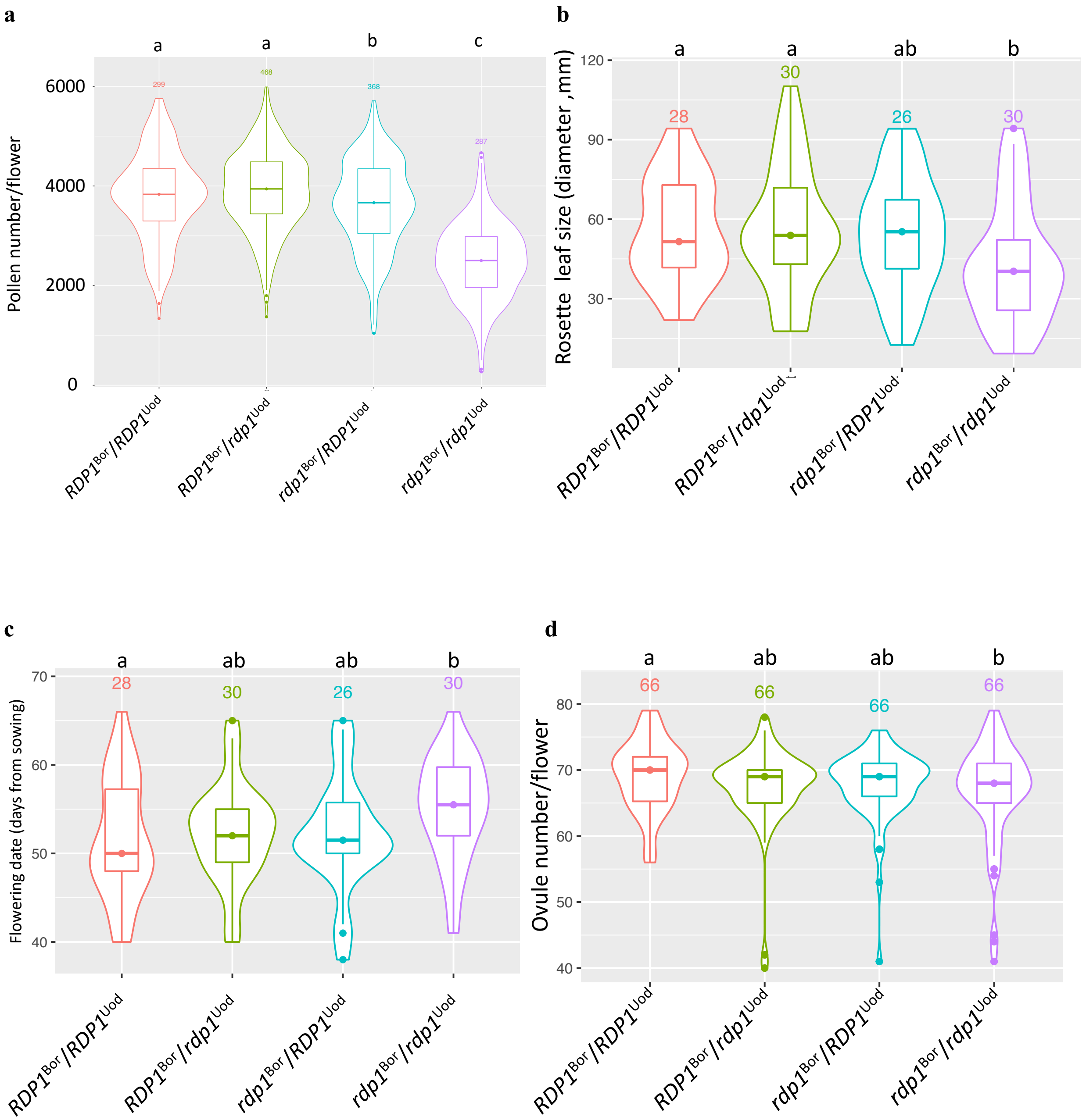

a
a a b c
299 468 368 287
Pollen number/flower
0
2000
4000
6000
RDP1^Bor/RDP1^Uod
RDP1^Bor/rdp1^Uod
rdp1^Bor/RDP1^Uod
rdp1^Bor/rdp1^Uod
b
a a ab b
28 30 26 30
Rosette leaf size (diameter ,mm)
30
60
90
120
RDP1^Bor/RDP1^Uod
RDP1^Bor/rdp1^Uod
rdp1^Bor/RDP1^Uod
rdp1^Bor/rdp1^Uod
c
a ab ab b
28 30 26 30
Flowering date (days from sowing)
40
50
60
70
RDP1^Bor/RDP1^Uod
RDP1^Bor/rdp1^Uod
rdp1^Bor/RDP1^Uod
rdp1^Bor/rdp1^Uod
d
a ab ab b
66 66 66 66
Ovule number/flower
40
50
60
70
80
RDP1^Bor/RDP1^Uod
RDP1^Bor/rdp1^Uod
rdp1^Bor/RDP1^Uod
rdp1^Bor/rdp1^Uod

e f

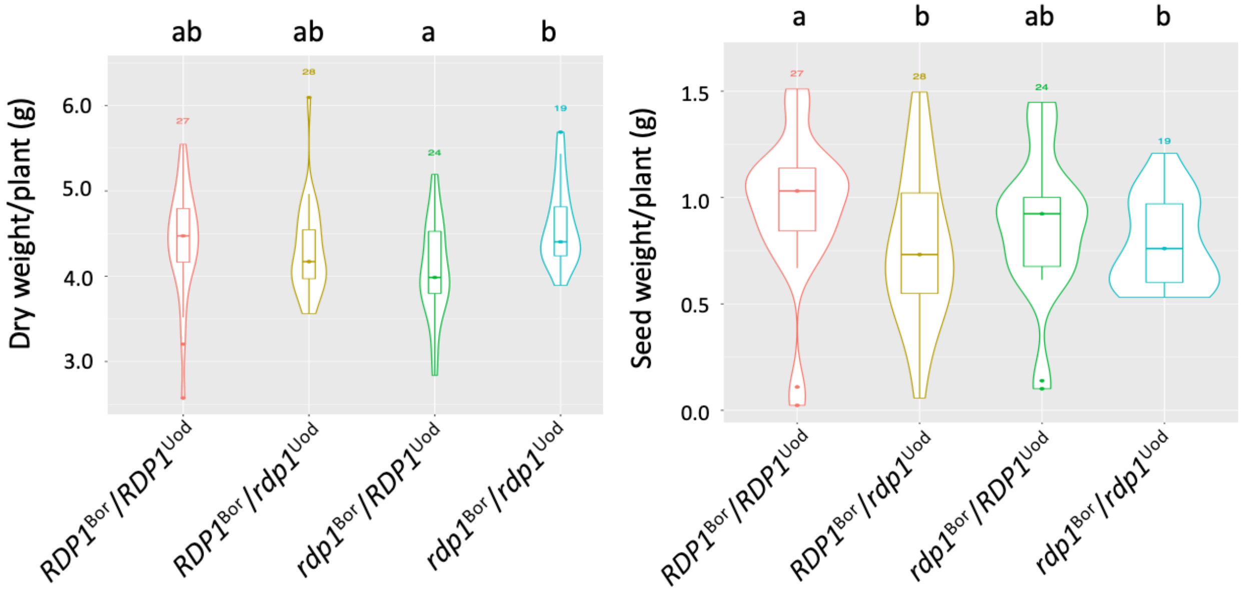

Supplementary Figure 17. Phenotypes of F_1 plants obtained from the cross of *RDP1*^{Bor}*/rdp1*^{Bor} and *RDP1*^{Uod}*/rdp1*^{Uod}.
Pollen number (**a**), rosette leaf size (**b**), flowering date (days from sowing) (**c**), ovule number (**d**), dry weight (**e**) and seed weight (**f**) in *RDP1*^{Bor}/*RDP1*^{Uod}, *RDP1*^{Bor}/*rdp1*^{Uod}, *rdp1*^{Bor}/*RDP1*^{Uod}, *rdp1*^{Bor}/*rdp1*^{Uod} plants. Sample numbers are shown above the plots. *P* values were listed in Supplementary Table 12. Letters (a, b, c) indicate significant differences determined by *t*-test; $P < 0.05$. For pollen number phenotype, the same data set was partially used with Fig. 3c. Boxplots show center line: median; box limits: upper and lower quartiles; whiskers: not greater than 1.5 times the interquartile range; point: outlier. Violin shape corresponds to the density of data. Source data are provided as a Source Data file.

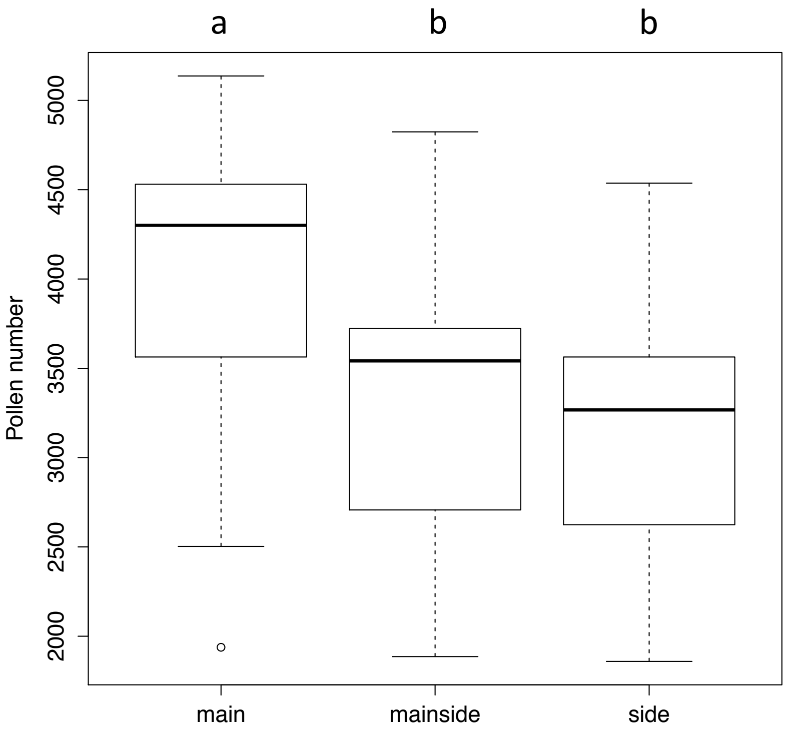

Supplementary Figure 18. Pollen number in different floral stems.
Main stems (main, N = 19), side stems branched from main stems (mainside, N = 20), and side stems (side, N = 19). Col-0 plants were used. Letters (a and b) indicate significant differences in pollen number between positions determined by analysis of variance and *post hoc* Tukey test ($P < 0.05$; main vs. mainside: $P = 0.0160$; main vs. side: $P = 0.00255$; mainside vs. side: $P = 0.769$). Boxplots show center line: median; box limits: upper and lower quartiles; whiskers: not greater than 1.5 times the interquartile range; point: outlier. Source data are provided as a Source Data file.

Supplementary Table 1. Pollen number phenotype of 144 accessions used for GWAS.

ecotype_id	Pollen number	ecotype_id	Pollen number	ecotype_id	Pollen number
5837	7355.416	7201	3420.000	7337	3276.000
6041	4459.114	7205	5904.000	7340	4068.000
6108	4219.922	7210	2808.000	7343	4032.000
6189	5265.087	7223	3168.000	7344	3384.000
6193	4626.375	7224	7632.000	7351	3888.000
6903	8549.642	7227	2952.000	7353	6696.000
6907	3871.534	7228	4608.000	7354	2880.000
6909	5508.000	7229	3924.000	7355	4680.000
6911	3024.000	7231	5544.000	7372	2844.000
6915	3904.466	7238	3708.000	7373	5256.000
6929	3280.487	7239	3816.000	7376	5760.000
6931	5265.087	7242	4536.000	7378	4104.000
6933	3488.480	7244	3600.000	7379	4464.000
6936	4043.128	7246	3528.000	7381	2592.000
6937	4336.052	7252	3564.000	7382	4788.000
6938	3784.004	7255	4104.000	7384	5472.000
6939	5090.893	7256	3132.000	7386	5616.000
6940	2710.240	7258	2952.000	7390	5976.000
6943	4336.052	7260	2088.000	7392	3996.000
6945	3771.871	7262	2808.000	7394	3672.000
6951	3465.081	7265	4608.000	7403	5004.000
6956	3627.142	7268	4536.000	7404	3024.000
6957	4112.459	7275	3060.000	7405	4212.000
6958	4103.793	7276	3780.000	7406	3384.000
6959	3003.163	7277	2880.000	7408	4500.000
6960	4568.310	7280	3528.000	7411	2880.000
6961	3349.818	7282	2880.000	7413	3132.000
6963	3284.820	7283	5652.000	7418	3240.000
6966	4219.922	7284	4176.000	7423	4032.000
6967	3929.599	7287	2772.000	7424	5472.000
6970	3384.484	7291	2304.000	7425	4356.000
6972	4667.107	7292	4176.000	7430	3456.000
6975	2594.110	7297	3816.000	7438	3067.294
6976	3523.146	7299	4644.000	7446	3600.000
6977	4452.181	7300	3240.000	7449	3888.000
6979	3407.016	7301	5040.000	7457	4392.000
6980	4452.181	7303	5112.000	7514	4944.431
6982	4112.459	7305	3996.000	7515	3000.563
6984	5126.425	7307	2880.000	7520	3465.081
6985	4452.181	7309	5544.000	7521	2942.499
7169	5256.000	7310	4356.000	7523	3290.887
7172	4068.000	7316	4644.000	7525	3037.829
7176	3636.000	7317	2844.000	8258	3290.887
7178	2808.000	7320	5832.000	8608	3581.210
7188	2124.000	7328	3096.000	8730	2926.033
7192	3744.000	7330	2304.000	8770	2587.177
7195	4140.000	7331	3888.000	8787	4045.728
7199	3204.000	7333	4104.000	8791	4219.922

Supplementary Table 2. Ovule number phenotype of 151 accessions used for GWAS.

ecotype_id	Ovule number	ecotype_id	Ovule number	ecotype_id	Ovule number
5837	64.000	7192	78.667	7333	86.667
6041	87.556	7195	77.200	7337	79.600
6108	78.667	7199	66.250	7340	84.667
6189	74.000	7201	69.500	7343	71.500
6193	76.500	7205	66.000	7344	74.667
6903	85.333	7210	80.000	7352	50.000
6907	65.500	7223	74.000	7353	58.500
6908	64.800	7224	75.250	7354	56.500
6909	66.500	7227	60.750	7355	57.500
6911	71.000	7228	70.667	7372	69.500
6915	76.000	7229	61.000	7373	75.500
6928	54.000	7231	74.800	7376	60.000
6929	72.500	7238	56.500	7378	69.000
6931	79.000	7239	69.500	7379	63.500
6933	64.800	7242	62.000	7381	65.750
6936	81.067	7244	76.000	7382	70.750
6937	73.250	7246	66.000	7384	51.500
6938	65.000	7252	47.333	7386	65.800
6939	76.000	7255	73.750	7390	50.250
6940	59.429	7256	62.500	7391	47.000
6943	79.000	7258	66.000	7392	72.000
6944	84.000	7260	69.250	7394	70.000
6945	58.933	7262	72.000	7403	75.500
6951	68.286	7265	61.200	7404	64.000
6956	61.429	7268	78.667	7405	71.500
6957	64.000	7275	85.000	7406	68.000
6958	77.333	7277	55.500	7408	72.000
6959	73.750	7280	63.500	7411	65.500
6960	75.500	7282	71.000	7413	68.000
6961	70.286	7283	56.500	7418	61.600
6963	76.000	7284	55.000	7423	72.000
6966	81.538	7287	60.000	7424	66.500
6967	69.000	7291	52.500	7425	71.500
6970	65.500	7292	70.000	7430	76.750
6972	73.667	7296	74.000	7438	67.000
6975	71.000	7297	70.667	7446	50.000
6976	61.667	7299	75.000	7449	66.000
6977	68.667	7300	57.000	7457	71.333
6978	66.667	7301	41.000	7514	70.000
6979	73.500	7303	60.667	7515	47.000
6980	73.500	7305	84.000	7520	84.727
6982	64.000	7307	68.000	7521	71.429
6984	86.000	7309	55.500	7523	56.500
6985	65.000	7310	83.600	7525	67.200
7169	44.333	7316	58.667	8258	75.500
7172	77.000	7317	65.800	8534	59.333
7176	69.500	7320	60.000	8608	60.000
7178	75.500	7328	49.000	8730	62.667
7181	52.000	7330	66.000	8770	65.333
7188	65.500	7331	62.750	8787	71.500
				8791	68.000

Supplementary Table 3. Association between pollen and ovule numbers and the diverse set of 107 phenotypes.

Category	Phenotype name	*r* (pollen)	*P* value (pollen)	*r* (ovule)	*P* value (ovule)
FL	1_LD	-0.8552	0.3974	-1.5384	0.1308
FL	2_LDV	-1.0132	0.3169	-1.8827	0.0661
FL	3_SD	-0.8687	0.3903	-2.0476	0.0466
FL	4_SDV	-0.0641	0.9492	-1.5905	0.1189
FL	5_FT10	-0.6123	0.5433	-0.4831	0.6310
FL	6_FT16	-1.0725	0.2891	-1.7206	0.0914
FL	7_FT22	-1.7315	0.0899	-3.3594	0.0015
DEV	8_Seed.Dormancy	-0.9639	0.3415	-0.2198	0.8271
DEF	9_Emco5	-1.0075	0.3204	-1.2389	0.2226
DEF	10_Emwa1	1.5968	0.1193	-0.0721	0.9429
DEF	11_Emoy.	1.8927	0.0667	-0.0061	0.9951
DEF	12_Hiks1	1.3100	0.1995	-0.5055	0.6163
DEF	13_Noco2	1.2426	0.2220	1.5227	0.1357
ION	14_Li7	2.1396	0.0389	3.0258	0.0042
ION	15_B11	-1.0237	0.3125	0.0339	0.9731
ION	16_Na23	0.3026	0.7638	-0.8206	0.4165
ION	17_Mg25	1.6761	0.1019	-0.3061	0.7610
ION	18_P31	-1.6564	0.1059	-0.1148	0.9091
ION	19_S34	0.5911	0.5580	0.4184	0.6778
ION	20_K39	0.0590	0.9532	-2.1852	0.0345
ION	21_Ca43	0.7194	0.4763	-0.6555	0.5157
ION	22_Mn55	-0.8659	0.3920	1.1553	0.2545
ION	23_Fe56	0.2868	0.7758	-1.8483	0.0716
ION	24_Co59	-0.7493	0.4583	-0.5890	0.5590
ION	25_Ni60	0.4532	0.6530	-0.0888	0.9296
ION	26_Cu65	0.0718	0.9431	0.6302	0.5320
ION	27_Zn66	0.6017	0.5509	-1.0796	0.2865
ION	28_As75	0.2697	0.7889	1.8220	0.0756
ION	29_Se82	0.9042	0.3716	0.6291	0.5327
ION	30_Mo98	-0.1156	0.9086	0.1483	0.8828
ION	31_Cd114	0.9132	0.3669	0.0092	0.9927
DEF	32_.i.avrPphB..i.	0.7417	0.4631	1.1303	0.2651
DEF	33_.i.avrRpm1..i.	0.9748	0.3365	1.2484	0.2195
DEF	34_.i.avrRpt2..i.	0.0851	0.9327	-0.4572	0.6500
DEF	35_.i.avrB..i.	1.0172	0.3157	1.3865	0.1731
FL	39_0W	-1.7238	0.0936	-4.5784	<0.0001

Category	Phenotype name	*r* (pollen)	*P* value (pollen)	*r* (ovule)	*P* value (ovule)
FL	40_2W	-1.2746	0.2106	-5.3055	<0.0001
FL	41_4W	-1.3665	0.1805	-5.2682	<0.0001
FL	42_8W	-1.5652	0.1260	-4.4811	0.0001
FL	43_FLC	-1.3672	0.1790	-0.7493	0.4575
FL	44_FRI	-0.5210	0.6052	-1.8254	0.0744
FL	45_8W.GH.FT	-0.9187	0.3636	-4.7241	<0.0001
FL	46_8W.GH.LN	-0.3272	0.7452	-3.1061	0.0032
FL	47_0W.GH.FT	-1.5063	0.1405	-3.8167	0.0004
FL	48_0W.GH.LN	-1.1528	0.2564	-3.4669	0.0012
FL	57_FT.Field	0.4327	0.6675	0.0373	0.9704
FL	58_FT.Diameter.Field	-1.4339	0.1592	-0.4120	0.6823
FL	59_FT.GH	-1.6428	0.1081	-3.1723	0.0027
DEV	60_FT.Duration.GH	-1.5166	0.1370	-1.6283	0.1103
DEV	61_LC.Duration.GH	-1.9732	0.0552	-3.0069	0.0043
DEV	62_LFS.GH	-1.8465	0.0720	-3.1233	0.0031
DEV	63_MT.GH	0.7352	0.4664	2.0756	0.0435
DEV	64_RP.GH	-1.1985	0.2376	-0.3450	0.7317
DEF	65_At1	-0.2961	0.7687	-1.2011	0.2360
DEF	66_At1.CFU2	-0.4207	0.6762	0.9256	0.3596
DEF	67_As	-2.9794	0.0049	-0.9480	0.3482
DEF	68_As.CFU2	-0.1424	0.8875	-0.3565	0.7231
DEF	69_Bs	1.5872	0.1203	-0.8652	0.3915
DEF	70_Bs.CFU2	0.2052	0.8385	-0.0102	0.9919
DEF	71_At2	-1.2512	0.2181	-1.6334	0.1094
DEF	72_At2.CFU2	-0.6648	0.5100	-0.6018	0.5503
DEF	73_As2	-0.2429	0.8094	-0.7852	0.4365
DEF	74_As2.CFU2	0.9693	0.3382	1.3879	0.1720
DEV	75_FW	2.6348	0.0120	0.8764	0.3857
DEV	76_DW	2.1601	0.0370	1.0485	0.3003
DEV	77_LES	-1.0778	0.2877	0.3975	0.6929
DEV	78_YEL	-0.2012	0.8416	0.3321	0.7414
DEV	79_LY	-0.9568	0.3445	0.5497	0.5854
FL	80_LN10	-0.3843	0.7028	0.5851	0.5614
FL	81_LN16	-1.2748	0.2097	-2.1525	0.0368
FL	82_LN22	-1.7027	0.0962	-3.1208	0.0031
DEV	158_Silique.16	-0.3224	0.7489	1.4653	0.1501

Category	Phenotype name	*r* (pollen)	*P* value (pollen)	*r* (ovule)	*P* value (ovule)
DEV	159_Silique.22	-1.8674	0.0694	-0.6575	0.5143
DEV	161_Germ.10	1.2372	0.2231	-1.2910	0.2032
DEV	162_Germ.16	1.3593	0.1817	-0.0174	0.9862
DEV	163_Germ.22	-1.0055	0.3205	-0.6597	0.5127
DEV	164_Width.10	-0.9511	0.3471	1.1540	0.2544
DEV	165_Width.16	0.5501	0.5853	1.8152	0.0762
DEV	166_Width.22	1.0230	0.3123	1.0241	0.3112
DEV	167_Chlorosis.10	-0.7454	0.4603	-1.0094	0.3181
DEV	168_Chlorosis.16	-0.6342	0.5296	-1.2568	0.2153
DEV	169_Chlorosis.22	1.5453	0.1300	1.8458	0.0714
DEV	170_Anthocyanin.10	-0.9742	0.3357	0.9999	0.3226
DEV	171_Anthocyanin.16	1.4269	0.1614	0.9579	0.3432
DEV	172_Anthocyanin.22	-0.9137	0.3662	1.7044	0.0951
DEV	173_Leaf.serr.10	0.4007	0.6908	1.1439	0.2589
DEV	174_Leaf.serr.16	1.2759	0.2093	0.6462	0.5214
DEV	175_Leaf.serr.22	-0.1472	0.8837	-0.6053	0.5480
DEV	176_Leaf.roll.10	0.1889	0.8511	0.0975	0.9228
DEV	177_Leaf.roll.16	0.5837	0.5627	-1.1581	0.2529
DEV	178_Leaf.roll.22	-0.1862	0.8532	0.8616	0.3934
DEV	179_Rosette.Erect.22	-0.2484	0.8051	-0.1686	0.8668
DEV	182_Hypocotyl.length	-0.7765	0.4424	-0.2015	0.8413
DEF	183_Trichome.avg.C	-2.1268	0.0398	-0.8471	0.4016
DEF	184_Trichome.avg.JA	-1.5666	0.1253	-0.4948	0.6232
DEF	185_Aphid.number	-0.1845	0.8546	-0.5621	0.5770
DEF	186_Bacterial.titer	0.5172	0.6079	0.4432	0.6599
DEV	272_Seedling.Growth	0.1026	0.9188	0.8743	0.3867
DEV	273_Vern.Growth	-0.1666	0.8686	-0.0107	0.9915
DEV	274_After.Vern.Growth	0.6230	0.5368	0.8083	0.4232
DEV	277_Secondary.Dormancy	2.2562	0.0304	1.6605	0.1048
DEV	278_Germ.in.dark	-2.1808	0.0360	-2.2820	0.0280
DEV	279_DSDS50	0.2367	0.8141	1.1101	0.2730
DEV	280_Seed.bank.133.91	0.1515	0.8804	0.8540	0.3978
DEV	281_Storage.7.days	-0.6102	0.5452	1.5378	0.1312
DEV	282_Storage.28.days	0.3415	0.7345	0.6413	0.5247
DEV	283_Storage.56.days	0.4940	0.6240	-0.8072	0.4239

FL: flowering phenotypes. DEV: developmental phenotypes. DEF: defense-related phenotypes. ION: ionomics phenotypes. For the details of phenotypes, see Atwell et al (ref. 2).

r: Pearson's correlation coefficient. *P* values were calculated by two-sided tests without adjustment for multiple comparison. For pollen number, log-transformed phenotype values were used.

Supplementary Table 4. *S*-haplogroups of the accessions used for pollen number GWAS.

ecotype_id	Accession name	*S*-haplogroup	ecotype_id	Accession name	*S*-haplogroup	ecotype_id	Accession name	*S*-haplogroup
5837	Bor-1	A	7201	Kr-0	C	7337	Si-0	A
6041	Lis-3	NA	7205	Krot-2	NA	7340	Sav-0	A
6108	T480	NA	7210	La-1	A	7343	Sp-0	A
6189	TDr-2	NA	7223	Li-2:1	A	7344	Sg-1	A
6193	TDr-7	NA	7224	Li-3	A	7351	Ty-0	NA
6903	Bor-4	A	7227	Li-5:2	NA	7353	Tha-1	NA
6907	CIBC-17	A	7228	Li-5:3	NA	7354	Ting-1	NA
6909	Col-0	A	7229	Li-6	A	7355	Tiv-1	NA
6911	Cvi-0	B	7231	Li-7	A	7372	Tscha-1	NA
6915	Ei-2	A	7238	Ll-1	A	7373	Tsu-0	NA
6929	Kondara	A	7239	Ll-2	C	7376	Tu-1	A
6931	Kz-9	A	7242	Lo-2	A	7378	Uk-1	A
6933	LL-0	A	7244	Mnz-0	NA	7379	Uk-2	A
6936	Lz-0	C	7246	Ma-2	A	7381	Uk-4	A
6937	Mrk-0	A	7252	Mc-0	NA	7382	Utrecht	NA
6938	Ms-0	A	7255	Mh-0	NA	7384	Ven-1	NA
6939	Mt-0	A	7256	Mh-1	A	7386	Vi-0	A
6940	Mz-0	A	7258	Nw-0	A	7390	Wag-3	NA
6943	NFA-10	A	7260	Nw-2	A	7392	Wag-5	NA
6945	Nok-3	A	7262	Nw-4	A	7394	Wa-1	A
6951	Pu2-23	A	7265	Nd-0	A	7403	Wei-1	A
6956	Pu2-7	A	7268	Np-0	NA	7404	Wc-1	A
6957	Pu2-8	NA	7275	No-0	A	7405	Wc-2	A
6958	Ra-0	C	7276	Ob-0	A	7406	Wt-1	A
6959	Ren-1	A	7277	Ob-1	NA	7408	Wt-3	NA
6960	Ren-11	A	7280	Old-1	A	7411	Wl-0	NA
6961	Se-0	A	7282	Or-0	A	7413	Wil-2	A
6963	Sorbo	A	7283	Ors-1	NA	7418	Zu-1	A
6966	Sq-1	A	7284	Ors-2	NA	7423	Jl-2	A
6967	Sq-8	A	7287	Ove-0	A	7424	Jl-3	A
6970	Ts-1	A	7291	Pa-2	A	7425	Jl-4	NA
6972	Tsu-1	A	7292	Pa-3	A	7430	Nc-1	A
6975	Uod-1	A	7297	Pf-0	A	7438	N13	NA
6976	Uod-7	A	7299	Pi-2	A	7446	N4	NA
6977	Van-0	A	7300	Pla-0	A	7449	N7	NA
6979	Wei-0	A	7301	Pla-1	A	7457	Rld-2	A
6980	Ws-0	A	7303	Pla-3	A	7514	RRS-7	NA
6982	Wt-5	C	7305	Pt-0	A	7515	RRS-10	NA
6984	Zdr-1	A	7307	Pn-0	A	7520	Lp2-2	A
6985	Zdr-6	A	7309	Po-1	A	7521	Lp2-6	A
7169	Hh-0	NA	7310	Pr-0	A	7523	Pna-17	NA
7172	Hl-3	A	7316	Rhen-1	NA	7525	Rmx-A180	NA
7176	Is-1	A	7317	Ri-0	NA	8258	Bå4-1	NA
7178	Jm-1	A	7320	Rou-0	A	8608	11ME1.23	NA
7188	Kelsterbach-2	A	7328	Sf-2	A	8730	11PNA1.9	NA
7192	Kil-0	A	7330	Sapporo-0	NA	8770	11PNA3.65	NA
7195	Kl-1	A	7331	Sh-0	A	8787	11PNA3.86	NA
7199	Kl-5	A	7333	Sei-0	A	8791	11PNA3.90	NA

The *S*-haplogroup assignment was based on Shimizu et al (ref. 1).
NA: not known.

Supplementary Table 5. Association between pollen and ovule numbers and climate variables.

	r (pollen)	*P* value (pollen)	*r* (ovule)	*P* value (ovule)
LATITUDE	0.0252	0.9799	0.4996	0.6182
LONGITUDE	0.4021	0.6883	1.3283	0.1865
bio4.temperature.seasonality	-0.0660	0.9475	1.3853	0.1684
bio5.maximum.temperature.in.the.warmest.month	-0.2095	0.8344	-0.9161	0.3613
bio6.minimum.temperature.in.the.coldest.month	-0.0695	0.9447	-2.2712	0.0248
bio13.precipitation.in.the.wettest.month	-1.2478	0.2145	-1.0581	0.2920
bio14.precipitation.in.the.driest.month	-0.3502	0.7268	-0.5938	0.5537
bio15.precipitation.cv	0.0513	0.9592	1.0617	0.2904
PAR_SPRING	0.3633	0.7170	-1.5635	0.1204
length.of.the.growing.season	0.1641	0.8699	-1.2036	0.2310
number.of.consecutive.cold.days	-0.2329	0.8162	0.8765	0.3824
number.of.consecutive.frost.free.days	-0.0637	0.9493	-2.9642	0.0036
relative.humidity_spring	-0.5172	0.6060	0.3048	0.7610
daylength.spring	-0.2057	0.8374	-0.0372	0.9704
aridity.index	-0.5043	0.6150	-1.7755	0.0782

For the details of climate variables, see Hancock et al (ref. 6).

r: Pearson's correlation coefficient (two-sided test without adjustment for multiple comparison).

For pollen number, log-transformed phenotype values were used.

Supplementary Table 6. Detailed SNP information of top five GWAS peaks with the 10-kb window-based iHS scores.

Chromosome	Position	GWAS score (-log10(*P* value))	MAF	iHS score	*P* value of iHS*
1	8858371	2.53E-08	0.167	-3.792	0.015
1	23683182	5.89E-08	0.160	2.398	0.359
2	10391465	4.58E-07	0.264	-3.561	0.026
3	15598975	1.32E-06	0.486	-1.608	0.873
5	101835	4.38E-06	0.424	1.695	0.829

**P* values of strongest iHS values in 10-kb windows that encompass the GWAS-SNP (one-sided test).

Supplementary Table 7. Top rank traits showing fold enrichments of more than 3 in the selection scan.

Phenotype name	Phenotype category	Short description	Fold enrichment	*P* value
Pollen number	This study	Pollen number per flower	6.35	0.002
Manganese (Mn55)	Ionomics	*In planta* ion concentration of Manganese	5.88	0.006
Bs CFU2	Defense-related	*In planta* bacterial growth (number of CFU / leaf area) of the *Pseudomonas viridiflava* strain RMX3.1b	5.77	0.003
Leaf roll 22	Developmental	Presence or absence of rolled leaves at 8 weeks post germination for plants grown at 10◦ C	5.13	0.001
LN10	Flowering	Leaf number at flowering time, 10˚C	4.71	0.004
LN16	Flowering	Leaf number at flowering time, 16˚C	4.44	0.006
Aphid number	Defense-related	Aphid offspring	4.44	0.008
RP GH	Developmental	Number of days between the appearance of the first flower and the plant complete senescence	4.26	0.009
LN22	Flowering	Leaf number at flowering time, 22˚C	4.17	0.007
Storage 28 days	Developmental	Primary dormancy	4.00	0.015
As	Defense-related	Disease presence or absence following inoculation with *Pseudomonas viridiflava* strain RMX23.1b	3.92	0.022
Nickel (Ni60)	Ionomics	*In planta* ion concentration of Nickel	3.85	0.019
Noco2	Defense-related	Disease presence or absence following inoculaton with *Perenospora parasitica* isolate Noco2	3.77	0.017
Ovule number	This study	Ovule number per flower	3.64	0.03
Molybdenum (Mo98)	Ionomics	*In planta* ion concentration of Molybdenum	3.57	0.03
Germ 10	Developmental	Days from removal from stratification until emergence of first cotyledon at 10˚C	3.27	0.018

Pollen and ovule numbers, and phenotypes of Atwell et al. (ref. 2) are shown.

Phenotype categories are based on Atwell et al (ref. 2). Fold enrichment and *P* values of the top 1% iHS tail are shown (one-sided test).

Supplementary Table 8. Pairwise *P* values of Fig. 2a.

Pairwise genotype combinations		*P* value
RDP1/RDP1	*rdp1-1/rdp1-1*	<2.2e-16
rdp1-1/rdp1-1+RDP1	*rdp1-1/rdp1-1*	<2.2e-16
rdp1-3/rdp1-3	*rdp1-1/rdp1-1*	0.183
rdp1-4/rdp1-4	*rdp1-1/rdp1-1*	<2.2e-16
rdp1-2/rdp1-2	*rdp1-1/rdp1-1*	0.173
rdp1-1/rdp1-1+RDP1	*RDP1/RDP1*	0.894
rdp1-3/rdp1-3	*RDP1/RDP1*	<2.2e-16
rdp1-4/rdp1-4	*RDP1/RDP1*	<2.2e-16
rdp1-2/rdp1-2	*RDP1/RDP1*	<2.2e-16
rdp1-3/rdp1-3	*rdp1-1/rdp1-1+RDP1*	<2.2e-16
rdp1-4/rdp1-4	*rdp1-1/rdp1-1+RDP1*	<2.2e-16
rdp1-2/rdp1-2	*rdp1-1/rdp1-1+RDP1*	<2.2e-16
rdp1-4/rdp1-4	*rdp1-3/rdp1-3*	0.982
rdp1-2/rdp1-2	*rdp1-3/rdp1-3*	0.043
rdp1-2/rdp1-2	*rdp1-4/rdp1-4*	0.0013

P values were calculated by two-sided tests with adjustments for multiple comparisons (post hoc Tukey test).

Supplementary Table 9. Pairwise *P* values of Supplementary Fig. 9.

Pairwise genotype combinations		*P* value
RDP1/RDP1	*rdp1-1/rdp1-1*	2.88E-04
rdp1-1/rdp1-1+RDP1	*rdp1-1/rdp1-1*	2.06E-05
rdp1-3/rdp1-3	*rdp1-1/rdp1-1*	0.999
rdp1-4/rdp1-4	*rdp1-1/rdp1-1*	2.86E-02
rdp1-2/rdp1-2	*rdp1-1/rdp1-1*	5.28E-05
rdp1-1/rdp1-1+RDP1	*RDP1/RDP1*	0.998
rdp1-3/rdp1-3	*RDP1/RDP1*	0.0266
rdp1-4/rdp1-4	*RDP1/RDP1*	0.999
rdp1-2/rdp1-2	*RDP1/RDP1*	<2.2e-16
rdp1-3/rdp1-3	*rdp1-1/rdp1-1+RDP1*	0.024
rdp1-4/rdp1-4	*rdp1-1/rdp1-1+RDP1*	0.999
rdp1-2/rdp1-2	*rdp1-1/rdp1-1+RDP1*	<2.2e-16
rdp1-4/rdp1-4	*rdp1-3/rdp1-3*	0.137
rdp1-2/rdp1-2	*rdp1-3/rdp1-3*	4.90E-03
rdp1-2/rdp1-2	*rdp1-4/rdp1-4*	<2.2e-16

P values were calculated by two-sided tests with adjustments for multiple comparisons (post hoc Tukey test).

Supplementary Table 10. Primer list.

Target	Purpose	Forward primer		Reverse primer	
		Primer name	sequence (5'-3')	Primer name	sequence (5'-3')
AT1G25250	quantitative reverse transcription PCR	3931_At1g25250RTf1	ACACCAGCTAACAGATCCAGATG	3932_At1g25250RTr1	AGATCTCACACACGTACCGATCTG
AT1G25260 (*RDP1*)	quantitative reverse transcription PCR	3933_At1g25260RTf1	AGCGAGATAGACCAGTGACTTTGTC	3934_At1g25260RTr1	CAACAGCCTCCCTAATTCCATTCAC
AT1G25270	quantitative reverse transcription PCR	4075_At1g25270RTf1	TTCGGATCATGACTGGGAGCAATG	4076_At1g25270RTr1	CGAAACCACGATTCCCGAATAAAGC
AT5G60390 (*EF1-α*)	quantitative reverse transcription PCR	3529_AhEF1aF	TGAGCACGCTCTTCTTGCTTTCA	3530_AhEF1aR	GGTGGTGGCATCCATCTTGTTACA
AT1G25250	genotyping for GT_5_108298 (wild-type)	3100_GT_5_107916_LP	AGACATGTTGGCTCAGGACAC	3101_GT_5_107916_RP	ATGTAAGGTGCGAGGTCAATG
	genotyping for GT_5_108298 (mutant)	3100_GT_5_107916_LP	AGACATGTTGGCTCAGGACAC	3264_Ds3-1	ACCCGACCGGATCGTATCGGT
AT1G25260 (*RDP1*)	genotyping for *rdp1-1* (wild-type)	3619_At1g25260F04	TATGATACCCCAATTGAATG	3195_SA_064854_RP	TTTCCATTGTGCCTAAACTGC
	genotyping for *rdp1-1* (mutant)	2939_SALK_T_DNA_LB	ATTTTGCCGATTTCGGAAC	3195_SA_064854_RP	TTTCCATTGTGCCTAAACTGC
	genotyping for *rdp1-2* (wild-type)	3622_At1g25260F07	TTGTAGTTTGTGAAGAAGG	3551_At1g25260R01	GTTTAAAATGAGAGAACCCG
	genotyping for *rdp1-2* (mutant)	3622_At1g25260F07	TTGTAGTTTGTGAAGAAGG	2946_GABI_T_DNA_LB	ATATTGACCATCATACTCATTGC

Supplementary Table 11. Accession numbers for Supplementary Figs 11 and 12.

Gene family	Species	Accession number or AGI code
RDP1/Mrt4p	*Arabidopsis thaliana* (Col-0 accession)	AT1G25260
	Arabidopsis thaliana (Mz-0 accession)	LC164158
	Arabidopsis thaliana (Bor-4 accession)	LC164159
	Arabidopsis thaliana (Uod-1 accession)	LC504218
	Arabidopsis lyrata	LC164160
	Arabidopsis halleri	LC164161
	Capsella rubella	XP006304175
	Theobroma cacao	XP007041559
	Populus trichocarpa	XP002299012
	Vitis vinifera	XP002276595
	Solanum lycopersicum	XP004230519
	Prunus persica	XP007223904
	Lotus japonicus	AFK33945
	Glycine max	NP001235726
	Medicago truncatula	XP003593096
	Oryza sativa	XP015618389
	Zea mays	NP001148655
	Physcomitrella patens	XP024389099
	Acanthamoeba castellanii	XP004351551
	Caenorhabditis elegans	NP495470
	Latimeria chalumnae	XP005986121
	Homo sapiens	AF173378_1
	Astyanax mexicanus	XP007251523
	Drosophila virilis	XP002050865
	Drosophila melanogaster	NP610554
	Saccharomyces cerevisiae	AJS35570
P0 protein	*Arabidopsis thaliana*	AT3G09200
	Arabidopsis thaliana	AT3G11250
	Arabidopsis thaliana	AT2G40010
	Arabidopsis lyrata	EFH58973
	Arabidopsis halleri	LC164162
	Arabidopsis halleri	LC164163
	Drosophila melanogaster	NP524211
	Homo sapiens	NP000993
	Caenorhabditis elegans	NP492766
	Saccharomyces cerevisiae	NP013444
Yvh1	*Saccharomyces cerevisiae*	NP012292

Supplementary Table 12. *P* values for Supplementary Fig. 17.

Pollen number shown in Supplementary Fig. 17a.

	$RDP1^{Bor}/RDP1^{Uod}$	$RDP1^{Bor}/rdp1^{Uod}$	$rdp1^{Bor}/RDP1^{Uod}$	$rdp1^{Bor}/rdp1^{Uod}$
$RDP1^{Bor}/RDP1^{Uod}$	-	-	-	-
$RDP1^{Bor}/rdp1^{Uod}$	0.06361	-	-	-
$rdp1^{Bor}/RDP1^{Uod}$	**0.01248**	**1.23E-06**	-	-
$rdp1^{Bor}/rdp1^{Uod}$	**< 2.2e-16**	**< 2.2e-16**	**< 2.2e-16**	-

Rosette leaf size shown in Supplementary Fig. 17b.

	$RDP1^{Bor}/RDP1^{Uod}$	$RDP1^{Bor}/rdp1^{Uod}$	$rdp1^{Bor}/RDP1^{Uod}$	$rdp1^{Bor}/rdp1^{Uod}$
$RDP1^{Bor}/RDP1^{Uod}$	-	-	-	-
$RDP1^{Bor}/rdp1^{Uod}$	0.9843	-	-	-
$rdp1^{Bor}/RDP1^{Uod}$	0.7395	0.7479	-	-
$rdp1^{Bor}/rdp1^{Uod}$	**0.0239**	**0.03439**	0.06804	-

Flowering date shown in Supplementary Fig. 17c.

	$RDP1^{Bor}/RDP1^{Uod}$	$RDP1^{Bor}/rdp1^{Uod}$	$rdp1^{Bor}/RDP1^{Uod}$	$rdp1^{Bor}/rdp1^{Uod}$
$RDP1^{Bor}/RDP1^{Uod}$	-	-	-	-
$RDP1^{Bor}/rdp1^{Uod}$	0.7504	-	-	-
$rdp1^{Bor}/RDP1^{Uod}$	0.8215	0.9439	-	-
$rdp1^{Bor}/rdp1^{Uod}$	**0.04641**	0.05926	0.07581	-

Ovule number shown in Supplementary Fig. 17d.

	$RDP1^{Bor}/RDP1^{Uod}$	$RDP1^{Bor}/rdp1^{Uod}$	$rdp1^{Bor}/RDP1^{Uod}$	$rdp1^{Bor}/rdp1^{Uod}$
$RDP1^{Bor}/RDP1^{Uod}$	-	-	-	-
$RDP1^{Bor}/rdp1^{Uod}$	0.1237	-	-	-
$rdp1^{Bor}/RDP1^{Uod}$	0.1591	0.8317	-	-
$rdp1^{Bor}/rdp1^{Uod}$	**0.04947**	0.549	0.4183	-

Dry weight shown in Supplementary Fig. 17e.

	$RDP1^{Bor}/RDP1^{Uod}$	$RDP1^{Bor}/rdp1^{Uod}$	$rdp1^{Bor}/RDP1^{Uod}$	$rdp1^{Bor}/rdp1^{Uod}$
$RDP1^{Bor}/RDP1^{Uod}$	-	-	-	-
$RDP1^{Bor}/rdp1^{Uod}$	0.5022	-	-	-
$rdp1^{Bor}/RDP1^{Uod}$	0.0608	0.154	-	-
$rdp1^{Bor}/rdp1^{Uod}$	0.2847	0.0571	**0.002835**	-

Seed weight shown in Supplementary Fig. 17f.

	$RDP1^{Bor}/RDP1^{Uod}$	$RDP1^{Bor}/rdp1^{Uod}$	$rdp1^{Bor}/RDP1^{Uod}$	$rdp1^{Bor}/rdp1^{Uod}$
$RDP1^{Bor}/RDP1^{Uod}$	-	-	-	-
$RDP1^{Bor}/rdp1^{Uod}$	**0.04554**	-	-	-
$rdp1^{Bor}/RDP1^{Uod}$	0.355	0.291	-	-
$rdp1^{Bor}/rdp1^{Uod}$	**0.04923**	0.877	0.3233	-

Bold *P* values are significant at $P < 0.05$ (two-sided test, post hoc Tukey test).

Supplementary References

1. Shimizu, K. K., Shimizu-Inatsugi, R., Tsuchimatsu, T. & Purugganan, M. D. Independent origins of self-compatibility in *Arabidopsis thaliana*. *Mol. Ecol.* **17,** 704–714 (2008).
2. Atwell, S. *et al.* Genome-wide association study of 107 phenotypes in *Arabidopsis thaliana* inbred lines. *Nature* **465,** 627–631 (2010).
3. Sicard, A. & Lenhard, M. The selfing syndrome: a model for studying the genetic and evolutionary basis of morphological adaptation in plants. *Ann. Bot.* **107,** 1433–1443 (2011).
4. Saitou, N. & Nei, M. The neighbor-joining method: a new method for reconstructing phylogenetic trees. *Mol. Biol. Evol.* **4**, 406–425 (1987).
5. Kimura, M. A simple method for estimating evolutionary rates of base substitutions through comparative studies of nucleotide sequences. *J. Mol. Evol.* **16**, 111–120 (1980).
6. Hancock, A. M. *et al.* Adaptation to climate across the *Arabidopsis thaliana* genome. *Science* **334,** 83–86 (2011).
