# Supplementary material for: Adaptive reduction of male gamete number in the selfing plant Arabidopsis thaliana
Source: Nat Commun. 2020 Jun 8;11:2885. doi: 10.1038/s41467-020-16679-7 (PMC7280297; doi:10.1038/s41467-020-16679-7)
Supplement: Supplementary file 3 — Description of Additional Supplementary Files [file 41467_2020_16679_MOESM3_ESM.pdf]

Description of Additional Supplementary Files

File name: Supplementary Data 1
Description: *Arabidopsis thaliana* accessions used in this study
